# LeGUI: A Fast and Accurate Graphical User Interface for Automated Detection and Anatomical Localization of Intracranial Electrodes

**DOI:** 10.3389/fnins.2021.769872

**Published:** 2021-12-09

**Authors:** Tyler S. Davis, Rose M. Caston, Brian Philip, Chantel M. Charlebois, Daria Nesterovich Anderson, Kurt E. Weaver, Elliot H. Smith, John D. Rolston

**Affiliations:** ^1^Department of Neurosurgery, University of Utah, Salt Lake City, UT, United States; ^2^Department of Biomedical Engineering, University of Utah, Salt Lake City, UT, United States; ^3^Department of Pharmacology and Toxicology, University of Utah, Salt Lake City, UT, United States; ^4^Department of Radiology, University of Washington, Seattle, WA, United States; ^5^Department of Biological Structure, University of Washington, Seattle, WA, United States

**Keywords:** MATLAB, anatomical localization, graphical user interface (GUI), electrocorticography (ECoG), software, stereotactic electroencephalography (SEEG), intracranial electrode localization

## Abstract

Accurate anatomical localization of intracranial electrodes is important for identifying the seizure foci in patients with epilepsy and for interpreting effects from cognitive studies employing intracranial electroencephalography. Localization is typically performed by coregistering postimplant computed tomography (CT) with preoperative magnetic resonance imaging (MRI). Electrodes are then detected in the CT, and the corresponding brain region is identified using the MRI. Many existing software packages for electrode localization chain together separate preexisting programs or rely on command line instructions to perform the various localization steps, making them difficult to install and operate for a typical user. Further, many packages provide solutions for some, but not all, of the steps needed for confident localization. We have developed software, Locate electrodes Graphical User Interface (LeGUI), that consists of a single interface to perform all steps needed to localize both surface and depth/penetrating intracranial electrodes, including coregistration of the CT to MRI, normalization of the MRI to the Montreal Neurological Institute template, automated electrode detection for multiple types of electrodes, electrode spacing correction and projection to the brain surface, electrode labeling, and anatomical targeting. The software is written in MATLAB, core image processing is performed using the Statistical Parametric Mapping toolbox, and standalone executable binaries are available for Windows, Mac, and Linux platforms. LeGUI was tested and validated on 51 datasets from two universities. The total user and computational time required to process a single dataset was approximately 1 h. Automatic electrode detection correctly identified 4362 of 4695 surface and depth electrodes with only 71 false positives. Anatomical targeting was verified by comparing electrode locations from LeGUI to locations that were assigned by an experienced neuroanatomist. LeGUI showed a 94% match with the 482 neuroanatomist-assigned locations. LeGUI combines all the features needed for fast and accurate anatomical localization of intracranial electrodes into a single interface, making it a valuable tool for intracranial electrophysiology research.

## Introduction

Intracranial recordings in neurosurgical patients provide an opportunity to study population-level neural activity in the human brain (Parvizi and Kastner, 2018; Pesaran et al., 2018). Intracranial research is often conducted in patients with medically refractory epilepsy who elect to undergo invasive clinical brain monitoring for seizure onset zone localization (Jobst et al., 2020). Electrodes are surgically implanted into various brain regions thought to be involved in the seizure network. Intracranial depth electrodes, subdural grids, or both may be implanted depending on a patient’s seizure presentation and preoperative workup, utilizing stereotactic electroencephalography (SEEG) and electrocorticography (ECoG), respectively. Once electrodes are implanted, the recorded neural activity offers clinical insight related to the seizure source. Thus, accurate postimplantation electrode localization is vital to study the activity associated with seizure initiation and propagation. Accurate localization of intracranial electrodes is also informative for inferring the function of non-epileptic brain areas when these patients participate in research studies. Prior intracranial studies have been important in understanding the neural computations underlying primary sensory and motor functions (Miller et al., 2007; Mesgarani and Chang, 2012; Bouchard et al., 2013; Hermes et al., 2015; Jiang et al., 2017), cognition (Jacobs et al., 2013; Smith et al., 2015; Bastuji et al., 2016; Minxha et al., 2020), and neurological and psychiatric disorders (Weiss et al., 2013; Martinet et al., 2017; Smith et al., 2020).

Numerous software packages have been designed to localize intracranial electrodes (Ekstrom et al., 2008; Hermes et al., 2010; Gramfort et al., 2013; Blenkmann et al., 2017; Groppe et al., 2017; Hamilton et al., 2017; Laplante et al., 2017; Qin et al., 2017; Branco et al., 2018; Granados et al., 2018; Trotta et al., 2018; Li et al., 2019). Most packages start by coregistering preimplantation magnetic resonance imaging (MRI) with postimplantation computed tomography (CT). Electrode locations are then derived from the CT images, and anatomical information is often obtained by registering an anatomically labeled atlas to the coregistered MRI. Many of these packages are built upon previously existing software, are written in various programming languages, and are sometimes only compatible with specific operating systems. This heterogeneity not only creates an issue of dependencies and increases the complexity of installation, but it can also decrease efficiency, resulting in longer computation and user times. For some packages, image processing can take up to 24 h (Groppe et al., 2017). Additionally, many of the existing packages require manual detection of individual electrodes (Hermes et al., 2010; Gramfort et al., 2013; Groppe et al., 2017; Trotta et al., 2018), adding to the overall user time and increasing the potential for errors. Most packages are only compatible with ECoG electrodes and have not been tested on SEEG electrodes (Hermes et al., 2010; Groppe et al., 2017; Branco et al., 2018; Trotta et al., 2018), which are increasingly prevalent in the United States (Abou-Al-Shaar et al., 2018). However, it is important to have the option to localize various types of electrodes, because the use of different electrodes may be required depending on clinical needs.

Here we present a software package, Locate electrodes Graphical User Interface (LeGUI), developed in the MATLAB programming language (Mathworks, Natick, MA), to localize and visualize intracranial electrodes. We designed LeGUI to be computationally efficient and easy to use, while retaining the functionality required for a typical intracranial neurophysiology study. Using MRI and CT images as an input, LeGUI generates and visualizes 2D and 3D models of the brain and electrode locations, including anatomical localizations from several atlases. LeGUI consists of an integrated user interface for all processing steps, including linear coregistration of CT to MRI, non-linear normalization of MRI to the Montreal Neurological Institute (MNI) ICBM152 template (Mazziotta et al., 2001), automated electrode detection, electrode spacing correction and projection to a surface, electrode labeling and channel assignment, anatomical targeting using several atlases, and saving of electrode data for downstream analysis. LeGUI has a unique split-screen design, showing 2D images and electrode locations on the left and the corresponding 3D representations on the right. Core image processing (coregistration, segmentation, and normalization) is performed using the Statistical Parametric Mapping toolbox (SPM12)^1^ (Friston et al., 2007). All other processing and visualizations are performed using standard MATLAB functions with parallelization for added speed. The software is provided open source under the GNU General Public License v3.0 with compiled executables for Windows, Mac, and Linux operating systems. The software and user documentation are available for download at https://github.com/Rolston-Lab/LeGUI.

## Materials and Methods

### Patients and Testing

Locate electrodes Graphical User Interface was tested and validated using MRI and CT imaging datasets from 48 patients undergoing intracranial monitoring for epilepsy at the University of Utah between 2015 and 2021 (Supplementary Table 1). Additional datasets from three patients were included from the University of Washington (Harborview Medical Center and Seattle Children’s Hospital). These datasets were used to test performance on imaging data acquired from a different institution, with the goal of generalizing across different scanner systems, protocols, and electrodes. For each patient, the preimplant MRI and high-resolution postimplant CT were processed, which included rotation of the MRI to anterior/posterior commissure (ACPC) space, linear coregistration of the CT to the MRI, and normalization of the patient MRI to MNI space. A block diagram of the image processing steps is provided in Supplementary Figure 1. All testing was performed on a PC running Windows 10 Professional with two Xeon X5680 processors at 3.33 GHz, 128 GB of random-access memory, and a NVIDIA GTX 1060 graphics card.

This study was approved by the University of Utah Institutional Review Board.

### Electrodes

A total of 5089 (4695 Utah, 394 Washington) ECoG and SEEG electrode contacts were detected, labeled, and projected to the brain surface (ECoG) or corrected so that the intercontact spacing precisely followed the lead geometry (SEEG). The specific use of depth leads or grid arrays depended on the clinical need and, regarding electrode vendor, institutional availability. The majority of the ECoG electrodes were Ad-Tech grids or strips with 1-cm spacing and 4-mm diameter (2.3 mm exposed) electrode contacts (Ad-Tech, Racine, WI, United States) (Supplementary Table 2). However, one patient (P27) had a mini grid with an intercontact spacing of 3 mm and diameter of 2 mm. Another patient (P9) had a grid with an intercontact spacing of 7 mm and diameter of 4 mm (2.3 mm exposed). The SEEG electrodes were from three different vendors (Ad-Tech; Dixi Medical, Chaudefontaine, France; and PMT Corporation, Chanhassen, MN, United States) with different contact sizes and spacings (Supplementary Table 2). Most of the Ad-Tech SEEG electrodes had contact diameters of 0.86 mm, lengths of 2.29 mm, and an intercontact spacing of 5 mm. The Ad-Tech SEEG electrodes also included Behnke-Fried hybrid leads that contain both macro- and microwire electrodes. The macroelectrodes had contact diameters of 1.28 mm, lengths of 1.57 mm, and spacings of 5 mm. The Behnke-Fried microelectrodes were excluded from testing because they are typically too small to resolve clearly on postoperative CTs. Dixi SEEG electrodes all had the same contact diameter and length of 0.8 mm and 2 mm, respectively. Most of the intercontact spacings were 3.5 mm. However, some leads had multiple groups of 5 contacts with standard 3.5-mm intercontact spacing and intergroup spacings of 7 mm. The PMT SEEG electrodes from the University of Washington patients (P49-51) all had the same contact diameter, length, and spacing of 0.8, 2, and 3.5 mm, respectively. All electrodes were platinum/iridium. Overall, 830 Ad-Tech ECoG, 64 Ad-Tech mini ECoG, 2098 Ad-Tech SEEG, 200 Ad-Tech Behnke SEEG, 1503 Dixi SEEG, and 394 PMT SEEG electrode contacts were processed using LeGUI.

### User Interface

**FIGURE 1:**
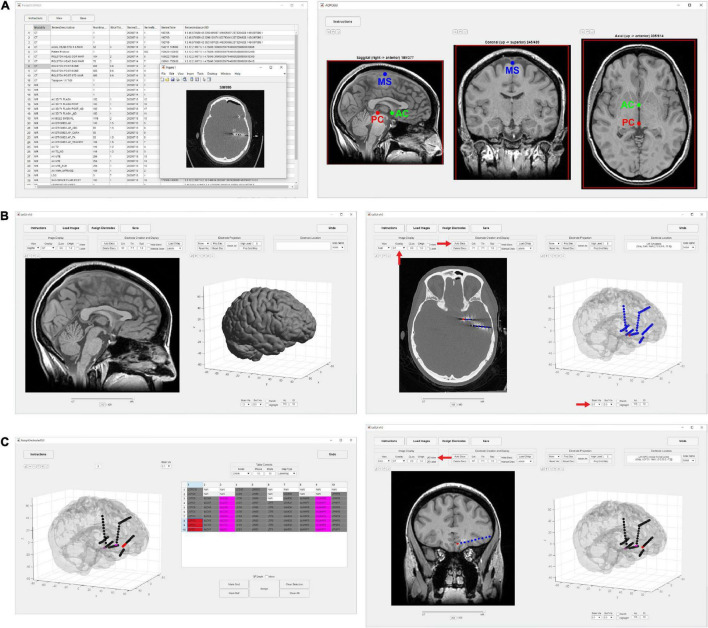
Flow chart of processing steps. **(A)** Image selection and saving (left) with ACPC alignment for MRI images (right). AC, PC and mid-sagittal (MS) points are shown in green, red, and blue, respectively. **(B)** Results after coregistration, segmentation, and MNI normalization steps (left). Gray and white segmented tissue types are used to generate a 3D brain surface. Automatic electrode detection results using coregistered CT (right). The opacity of the MRI image can be decreased to show the CT image with superimposed electrodes. The brain surface can be made transparent or hidden to visualize electrodes in 3D display. **(C)** User interface for electrode labeling and channel assignment (left). Assignments are color coded for visual clarity. Selected table values and corresponding assigned electrodes are shown in red. Assignments are populated into the main LeGUI interface after closing the assignment interface window (right). Inline projection of selected SEEG leads allows visualization of the entire lead relative to a single MRI slice plane.

Locate electrodes Graphical User Interface consists of a main window split between a 2D display of the MRI or CT image and electrode slice planes on the left and a 3D display of the brain surface and electrode locations on the right (Figure 1). Along the top of the window is a row of buttons (i.e., “Instructions,” “Load Images,” “Assign Electrodes,” “Save”) that initiate the main steps involved in processing a dataset. These buttons are arranged from left to right in sequence from the first to the last step. The leftmost “Instructions” button pulls up a list of step-by-step instructions. The “Load Images” button starts the initial processing steps of the MRI and CT images. The “Assign Electrodes” button opens a user interface for assigning labels and channel numbers to electrodes. The “Save” button saves electrode locations and assignments to a file in a folder named “Registered,” which is used by LeGUI to reopen previous datasets or can be used for later analysis.

### Image Loading and Anterior/Posterior Commissure Alignment

Initial image-processing steps begin with the “Load Images” button, which includes selecting and saving both the preimplant MRI and postimplant CT, coregistration of the CT to MRI, segmentation and normalization of MRI to MNI space, and creation of 3D brain and projection surfaces.

To begin, a directory containing both MRI and CT DICOM files must be specified. Files from multiple MRI and CT scans can reside in this directory. The software recursively searches for all images and displays the results in a table organized by modality, series number, series description, etc. (Figure 1A). The table is interactive and allows the user to view individual scans and save the desired scans as NIfTI^2^ files (“MR.nii” and “CT.nii”) in the “Registered” folder for later processing. The “Registered” folder is automatically created in the same root directory as the selected DICOM images once the first scan is saved. For optimal results, a standard preimplant T1-weighted MRI and postimplant CT with a slice thickness of 1 mm or less should be loaded. Images with any slice orientation (sagittal, coronal, or axial) can be loaded. LeGUI uses the standard right-anterior-superior (RAS) orientation convention where the positive x-axis points to the right, the positive y-axis points in the anterior direction, and the positive z-axis points in the superior direction. If images do not match this convention, they are automatically rotated to match, and the transform matrix is updated accordingly. If the automatic rotation fails, the option to manually rotate in increments of 90 degrees to match this orientation is also available. As images are loaded, an option to crop and rotate to ACPC space is provided. Cropping and ACPC rotation can be skipped for CT images, but it is recommended for MRI images. After CT and MRI images have been rotated and cropped, the MRI image is resampled to a resolution of 0.4 mm/voxel across all three dimensions to ensure consistent image rendering quality and performance across datasets. Resampling is performed using “imresize3.m” with cubic interpolation (MATLAB Image Processing Toolbox). The CT image is also resampled to the same resolution later as part of the coregistration step.

### Coregistration of Computed Tomography to Magnetic Resonance Imaging

Linear coregistration of the CT to MRI uses a 6-parameter (three translations and three rotations) rigid-body model and a normalized mutual information cost function to perform the registration (SPM12 function “spm_coreg.m” with default parameters). The CT is then transformed and resliced to match the MRI voxel-by-voxel and saved back to disk as “CT.nii.” Reslicing is performed using a 4th degree b-spline interpolation.

### Segmentation and Normalization to Montreal Neurological Institute Space

Segmentation of the MRI tissue planes and MNI normalization is performed using SPM12. A procedure called “unified segmentation” is performed that combines segmentation, bias correction, and spatial normalization into one model. Segmentation separates the image into six tissue classes: gray, white, cerebrospinal fluid, bone, soft tissue, and air/background. Bias correction removes any smoothly varying intensity differences across the image that are introduced by the MRI scanning itself. Spatial normalization generates “deformation fields” that describe the non-linear transform from patient space to MNI space. For the spatial normalization procedure, MNI-registered probability maps (“TPM.nii”) of the different tissue classes are used as priors to generate a non-linear deformation field that describes the best overlays of the tissue probabilities with the patient MRI image. To account for partial volume effects where a voxel is not a simple representation of one tissue type, each tissue class is modeled using one or more gaussians. Some steps are also implemented to clean up the tissue classes after segmentation. These include a simple Markov Random Field cleanup procedure and a routine that involves erosions and conditional dilations of the different tissue planes to clean up the borders and improve brain extraction. In general, LeGUI uses the SPM default parameters for most operations. However, for the unified segmentation step, a few changes have been made to improve consistency across datasets. In LeGUI, the number of gaussians for modeling the tissue classes is set to two for gray matter, white matter, cerebrospinal fluid, and air, three for bone, and four for soft tissue. The defaults in SPM are one for gray and white, two for cerebrospinal fluid and air, three for bone, and four for soft tissue. The sampling distance between points when estimating model parameters has also been increased slightly from the default value of 3 to 4. All other parameters are set to their respective defaults. The deformation fields generated during normalization to MNI space are automatically saved to the “Registered” folder and are used to apply atlas labels to the electrodes. Both the forward “y_MR.nii” and inverse “iy_MR.nii” deformation fields are saved.

### Generating Brain and Projection Surfaces

Both a 3D brain surface and a highly smoothed brain surface (projection surface) for correcting electrode positions after postsurgical brain shift are generated in LeGUI. Surface generation is performed using the custom function, “LeG_genSurfaces.m.” The function starts by thresholding (>0.95) the summed gray/white segmented images generated during the segmentation step. The resulting mask is cleaned by removing small, connected components that are separate from the main brain component. To create the brain surface, the mask is smoothed using a 3-mm cuboid kernel, and an isosurface (“isosurface.m”; MATLAB built-in function) is generated using a threshold value of 0.3. For the projection surface, the mask is more aggressively smoothed using a 10-mm cuboid kernel, and the isosurface is generated using the same threshold of 0.3. Small surface remnants (typically from the ventricles) that may be enclosed within the main surface are then removed using the density-based “dbscan” clustering algorithm (MATLAB Statistics and Machine Learning Toolbox). Remnants are removed by finding and deleting the corresponding vertices and faces of all but the largest cluster identified by dbscan.

### 2D and 3D Visualizations

After the image-processing steps are complete, LeGUI will be updated to display the resulting images and surfaces (Figure 1B, left). The 2D display on the left shows the overlaid slice planes from the coregistered MRI and CT images with electrode contacts embedded in each image after the detection process (see below). The 3D display on the right shows brain and projection surfaces and electrode locations relative to these surfaces. For the 2D display, slice plane orientation can be changed from the default “sagittal” view to either a “coronal” or “axial” view and scrolling of slices can be performed to traverse the entire image. For the 3D display, the surfaces and electrodes can be rotated for improved visualization of the electrode geometry. Pan and zoom functionality exist for both displays. In addition, electrodes can be selected in either display with a mouse click. Selecting an electrode in one display automatically selects and highlights the same electrode in the other display. This “linking” of the 2D and 3D displays during selection provides an efficient and useful way to explore the electrode space and visualize nearby brain structures. Anatomical locations and channel assignments are also shown for selected electrodes in the upper left corner of the main window. For more details regarding the visualizations and user interactions of LeGUI, see Supplementary Methods.

### Electrode Localization

Electrode centroids can be localized from intensities in the CT either automatically with a button press or manually with a right click over the CT artifact in the 2D display (Figure 1B, right). Automatic detection is performed using a custom function “LeG_AutoElecs.m.” This function searches for electrodes by finding connected components for a series of thresholds that are applied to the image. Twenty-one thresholds are tested that are evenly distributed across the 99th to 100th percentile range of Hounsfield intensity values for all voxels in the image. For each threshold, connected components are considered electrodes if they are located inside the projection surface and the volume is greater than 6 voxels and less than that of a sphere of radius 2 mm (∼33.5 mm^3^). After all thresholds have been tested, stable threshold regions are identified as uninterrupted segments where the number of detected electrodes does not change by more than five. An optimal threshold is chosen as the center threshold of the uninterrupted segment that contains the largest number of electrodes. A clean-up procedure is then performed to remove any detected electrodes that are less than 1 mm apart. Electrodes are sorted in descending order based on the mean intensity of the connected component, and only the top 250 electrodes are kept. A limit of 250 is placed on the total number of detected electrodes to avoid overloading the software and degrading performance. None of the patients included in this study had more than 250 electrode contacts implanted; however, this limit can easily be changed in the underlying code if more than 250 electrode contacts are implanted. The weighted centroid, calculated from the CT voxel intensities, of each connected component is used as the electrode center for display in LeGUI.

If automatic detection fails, electrodes can be manually localized with a right click of the mouse over the CT artifact in the 2D display. Electrodes are localized to the weighted centroid of the nearest connected component to the click point in the CT image or at the tip of the mouse cursor if the “Manual Draw” selection box is selected. Connected components are found by thresholding a small region (a cube that is six times the electrode radius) around the click point using a user-specified threshold value. We operationally defined detection rate or sensitivity as the ratio of the number of true electrode detections to the total number of implanted electrodes. False electrode detections can be easily removed by selecting the electrode with a left click and pressing either the “delete” or “backspace” key on the keyboard.

Once an electrode is localized, it is drawn in both the 2D and 3D displays. Each electrode is represented as a sphere with a default radius of 1.3 mm, which is a reasonable approximation of the radius of most intracranial electrodes; however, this value can be changed to better match the specific size of an electrode. Voxels that are enclosed within this radius are used to determine the atlas label and electrode class (gray or white) as described later.

### Electrode Labeling and Channel Assignment

After localizing electrodes, clicking on the “Assign Electrodes” button in LeGUI will open a user interface for assigning labels and channel numbers to electrodes (Figure 1C, left). This information is then used for some of the visualizations and functions in the main GUI window, but it can also be used to match electrode locations with neural recordings for later analysis. A 3D display of the electrodes with an overlay of the brain surface is provided to help with electrode identification. Assignments are then made by linking each 3D electrode with its corresponding label displayed in an alphanumeric table. Color-coding of assigned electrodes also makes navigation easier and helps with the identification of the remaining unassigned electrodes. Assignments of multiple electrodes can be made by selecting column-oriented groups in the table and the corresponding electrodes in the 3D display. This interface also has a “check” mode that allows the user to visually confirm the accuracy of the assignments before saving. For more details, see Supplementary Methods.

### Electrode Alignment and Projection

Locate electrodes Graphical User Interface offers both automated and manual methods for correcting the locations of electrodes if needed because of either brain shift from surgery or inaccurate centroid calculations during the automated detection process. For projecting ECoG grid and strip electrodes that were localized below the pial surface because of brain shift, an automated routine (Hermes et al., 2010) has been included. This routine projects electrodes onto a smoothed projection surface using the orthogonal local normal vector to the grid. For strips, where an orthogonal vector cannot be calculated, electrodes are projected to the nearest point on the surface. Electrode assignments need to be performed prior to using this projection method because labels are used to determine electrode neighbors for the orthogonal vector calculation.

As an alternative to automated projection, a manual method is included that does not require prior electrode assignments to be made. For this method, three projection vectors are calculated for each selected electrode, which can then be selected from a dropdown menu. The first vector points in the direction of the brain center (mid-commissural point) to the selected electrode. The second vector is in the direction of the current view of the 3D display so that it points out of the screen from the electrode center. The third vector is orthogonal to the projection surface at the point nearest to the selected electrode. Once a vector type has been chosen, vectors for each selected electrode are displayed as a red line projecting out from the electrode center to help visualize the trajectories. Electrodes can then be projected to the surface with a button press. Alternatively, the up/down arrows on the keyboard can be used to incrementally move selected electrodes along each vector path. This is useful for nudging electrodes outward if the brain surface partially obscures them or inward if electrodes have been projected too far and they fail to overlap with a nearby anatomical location.

A procedure for correcting spacing and alignment errors has also been included with LeGUI. This is typically applied to SEEG electrodes if irregularities in centroid spacing and alignment occurred during the detection step. This procedure uses electrode labels and the known intercontact spacing from the lead geometry to iteratively adjust the positions starting from the deepest electrode/contact and moving outward to the most superficial contact. Because labels are needed, the electrode assignments must be performed prior to this procedure. Also, the labels for each lead must be arranged as columns in the table in the electrode assignment user interface with the deepest electrode/contact as the last (bottom) row in each column. The deepest contact is used as the starting point because it typically is far away from contacts on other leads and has a more clearly defined intensity in the CT for the centroid calculation. Nearby crossing of leads and contacts that are close to the skull can cause a distorted CT artifact leading to a misplaced centroid and electrode/contact center. Before proceeding, the deepest contact should be visually inspected for accurate alignment with the CT artifact. This procedure starts by calculating the average vector between the deepest and 2nd and 3rd deepest contacts. The 2nd deepest contact is then repositioned using this vector so that its distance from the deepest contact is equal to the known intercontact spacing of the lead. This process is then repeated by repositioning the 3rd deepest contact using the average vector from the 2nd to the 3rd and 4th deepest contacts, and so on until the most superficial contact is reached. Upon completion of this repositioning step, the spacing between contacts is corrected to match the known, commercially defined spacing, and some subtle changes in contact position are made resulting in a straighter path of the contacts along a lead. Because this procedure operates along the columns of the table in the assignment interface, it could be applied to the electrode contacts of an ECoG grid or strip to correct the column-wise spacing if desired.

### Inline Projection

A unique feature called “inline projection” has been included in LeGUI to improve the visualization of SEEG electrodes relative to the images in the 2D display. This feature projects selected SEEG leads onto the current slice plane in the 2D display so that all contacts in a lead are visible in the same slice (Figure 1C, right). The corresponding MRI and CT images are rotated to match this projection line so that each contact along a lead can be visualized relative to MRI or CT structures. The vector made by the first and the last contact of a lead is used as the projection vector for calculating the rotation matrix that is applied to the MRI and CT images. Rotation is performed using the built-in “imwarp.m” function in MATLAB. Because leads are often oriented at an angle relative to the viewing plane, contacts along an individual lead cannot typically be viewed simultaneously. Therefore, without inline projection, these contacts can only be viewed individually by scrolling through the slice planes of the 2D image.

### Gray and White Electrode Classification

Each electrode/contact that has been created in LeGUI is classified as being in gray or white matter. This information is displayed in the main LeGUI window for selected electrodes and is saved to disk for later use in analysis. Class assignments are made by applying a two-sided Wilcoxon rank-sum test to the voxels enclosed within each electrode sphere taken from the gray and white tissue probability maps that are created during the SPM segmentation step. The rank sum tests the null hypothesis that the gray and white voxel probabilities are from continuous distributions with equal medians, against the alternative that they are not. If the null hypothesis is false (*p* < 0.001), assignments are made accordingly. If it is true, a default assignment of gray is given. If the mean voxel probability for an electrode is less than 0.1 for both gray and white, then an assignment of “Unknown” is given. These calculations are performed using the “LeG_calcGrayWhite.m” function.

### Anatomical Localizations and Atlas Overlays

Each electrode that has been created in LeGUI is assigned an anatomical location based on available atlases. The default atlases that are included with LeGUI are the Neuromorphometrics atlas (NMM)^3^ that comes bundled with SPM and the probabilistic cytoarchitectonic maps included in the SPM Anatomy toolbox (Eickhoff et al., 2005). To find the anatomical location and subsequent label, voxel indices for an electrode sphere in patient space are warped to MNI space by applying the SPM non-linear deformation fields in “iy_MR.nii.” Labels from the loaded MNI registered atlas are then found for each warped voxel location, and the most common label is assigned to that electrode. When saving, probabilities for all labels within a 1-cm radius around each electrode are also calculated and can be used later for analysis.

For visualization in LeGUI, available MNI-registered atlases are warped to patient space. This transformation is performed by applying the SPM MNI-to-patient deformation fields “y_MR.nii” to each voxel location in the labeled atlas NIfTI image. Atlases that are warped into patient space are saved to the main “Registered” folder and have the prefix “lw” (label warping) added to the file name. For example, the NMM atlas will have the name “lwNMM.nii.” Warped atlases can then be viewed in the 2D display in LeGUI as an overlay, with the ability to fade in and out with respect to the MRI. This provides a way to visually check the accuracy of the MNI normalization step.

In addition to the default atlases, custom atlases can be loaded into LeGUI by following a few simple steps. All custom atlases must be registered to MNI space. To load, the labeled NIfTI image can be placed in a folder named “atlases” that is in the same root directory as the LeGUI program. If LeGUI is run using the compiled binaries, then this folder must be in the same directory as the executable file. If LeGUI is run from the source code, then the “atlases” folder must be in the same directory as the “LeGUI.mlapp” file. In addition to the labeled atlas image, a tab-delimited text file containing the index numbers and corresponding label names must be included. Both the labeled image and text file must also have the same name. Once the “atlases” folder has been populated with these files, LeGUI will automatically detect the custom atlases and add them to a dropdown menu after the initial image-processing step.

### Saving

All data that is generated in LeGUI during the processing of images and electrodes is saved to the main “Registered” folder. This includes the MRI and CT images (“MR.nii” and “CT.nii”), the tissue segmentations (“MRGray.nii,” “MRWhite.nii,” “MRBone.nii,” “MRCSF.nii,” and “MRSkin.nii”), deformation fields from patient to MNI space and MNI to patient space (“iy_MR.nii” and “y_MR.nii”), atlases warped to patient space (“lwNMM.nii”), brain and projection surfaces (“surfaces.mat”), and all electrode information (“Electrodes.mat” and “ChannelMap.mat”). The images and surfaces are automatically generated during the image-processing steps. The electrode information is saved by pressing the “Save” button at the top of LeGUI after the detection and labeling steps have been performed.

The electrode files (“Electrodes.mat” and “ChannelMap.mat”) consist of MATLAB structures with fields that contain information about electrode location in millimeters for both patient and MNI space, anatomical location for each of the loaded atlases, and tissue class (gray or white matter). “Electrodes.mat” is primarily used by LeGUI to load electrode localization information for a previously processed dataset. This file can also be used for analysis. However, it is important to note that the data in this file is sorted based on the sequence of user interactions in LeGUI. Therefore, rows in each of the relevant data fields do not correspond to channel number. “ChannelMap.mat” contains electrode information that is sorted by channel number so that the row indices of each variable correspond to the channel number specified by the user during the assignment step. The “ChannelMap.mat” file is therefore preferred for use in analysis because electrode information is sorted to match the channel order of the recorded data. Details of all data that is included in these two files can be found in the “ReadMe.txt” file, which is automatically saved to the “Registered” folder during runtime. An example of the “ReadMe.txt” file is included in the Supplementary Material.

### Testing and Validation

Locate electrodes Graphical User Interface was used to process 48 datasets (4695 ECoG and SEEG electrode contacts) from patients undergoing long-term intracranial monitoring for epilepsy at the University of Utah. An additional three datasets from the University of Washington (394 SEEG electrode contacts) were also processed and were used to validate the anatomical labels generated by LeGUI.

Testing and validation were performed specifically to quantify processing times, automatic electrode detection, electrode projection and alignment, and anatomical localization. Most of the testing and validation was performed by T.D., the author of the software. Processing times were automatically collected for each dataset using built-in MATLAB timing functions. Automatic electrode detection was validated by comparing the automated results to the final detection results after removing false positives and manually adding electrodes that were missed by the algorithm. Electrode projection and alignment results were validated by comparing electrode locations before and after projection or alignment using Euclidean distance. Finally, anatomical localization was validated by comparing the labels generated by LeGUI to electrophysiology recordings as well as labels that were assigned by hand by a trained neuroanatomist (K.W.). Hand labeling was performed by coregistering the CT to the MRI through an affine registration and applying a mutual information cost function in FSL^4^. Electrode labels were then assigned by manually scrolling through overlaid slices of the two images and assigning labels specifically selected from the NMM atlas. An attempt was made to assign a label based on the center of the electrode artifact when visualized on the registered MRI scan. This was done in a blinded fashion such that no prior knowledge existed of the electrode locations or labels from LeGUI. Given the vast gyral complexity of human prefrontal and parietal association cortices in 2D, only temporal lobe, including medial temporal lobe structures and mid-line limbic regions, were manually labeled.

Other aspects of LeGUI, such as the quality of the coregistration and segmentation steps or aspects relating to user interface such as ease-of-use, were not quantified.

## Results

### Processing Times

**FIGURE 2:**
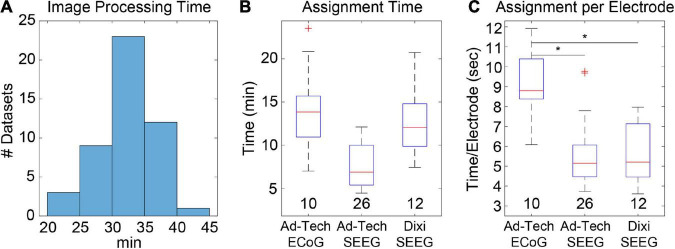
Image processing and electrode assignment times for 48 datasets in LeGUI. **(A)** Histogram of image processing times per dataset. Total time includes image selection, rotation to ACPC space, coregistration of CT to MRI, segmentation of MRI, normalization to MNI space, and surface generation. **(B)** Electrode label assignment times grouped by vendor and electrode type. Each boxplot shows the median (red line), interquartile range (blue box), and extremes (whiskers). The number of datasets per group is shown below each boxplot. **(C)** Electrode label assignment times normalized by number of electrodes per dataset. ECoG electrodes take a significantly (pairwise rank-sum test, *p* < 0.01) longer time to assign a label than SEEG. Asterisks indicate significance.

We measured elapsed times for the initial image-processing steps, which included image selection, rotation of MRI to ACPC space, coregistration of CT to MRI, segmentation of MRI into six tissue types, and normalization of MRI to MNI space. The median time and range for 48 datasets was 31.7 (21.9–40.3) min (Figure 2A). This included approximately 5–10 min of user time for selecting images and identifying AC, PC, and midsagittal points for rotation to ACPC space. The remaining time was computational and dedicated to the coregistration, segmentation, and normalization steps.

Automatic electrode detection times were measured and found to be small relative to the other processing steps. The median time and range for detection for 48 datasets (4695 total electrode contacts) was 15.6 (12.2–19.2) s. However, this does not include the user time required to correct for false-positive or false-negative detections. This time was not directly measured, but it is estimated to take approximately 5–10 min depending on the complexity of the implant and the performance of the automatic detection.

Most of the user time is devoted to electrode labeling and channel assignment. This step requires prior knowledge of the implanted electrodes based on patient-specific clinical notes and cannot be fully automated; however, the assignment interface in LeGUI has been designed to improve the efficiency of this step. Assignment times were measured for 48 datasets (4695 electrode contacts) (Figures 2B,C). The overall median time and range was 9.8 (4.5–23.6) min. Times were also divided into groups based on electrode type (Figure 2B). Assignment for Ad-Tech ECoG and Dixi SEEG took more time than for Ad-Tech SEEG, with median times and ranges of 13.9 (7–23.6), 12.1 (7.4–20.7), and 6.9 (4.5–12.1) min, respectively. However, after normalizing by the number of electrodes in each dataset, it was found that Ad-Tech ECoG assignment required significantly more time than assignment for both Dixi and Ad-Tech SEEG datasets (pairwise rank-sum test, *p* < 0.01), with a median time and range of 8.8 (6.1–12), 5.2 (3.6–8.0), and 5.1 (3.7–9.8) sec per electrode, respectively (Figure 2C). This is probably due to the increased complexity of these implants that often contain overlapping electrodes.

### Automatic Electrode Detection

**FIGURE 3:**
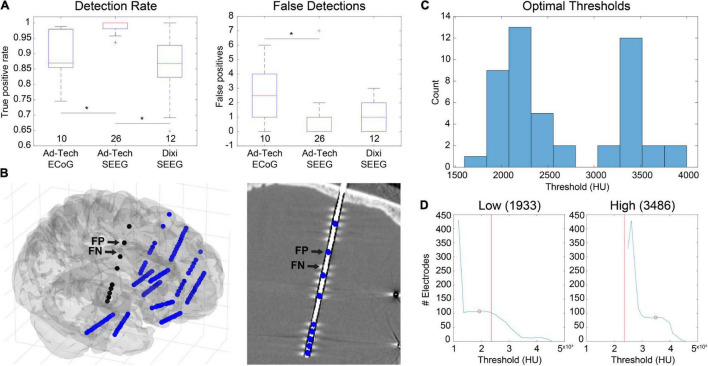
Automatic electrode detection results from 48 datasets containing 4695 total electrode contacts including ECoG and SEEG. **(A)** True-positive detections (left) and false positive detections (right) grouped by electrode type. Ad-Tech SEEG electrodes performed the best, with a median detection sensitivity of 1 and false-positive count of 0. Values under each boxplot indicate number of datasets included. Asterisks indicate significance (pairwise rank-sum, *p* < 0.01). **(B)** Example of false positives (FP) and false negatives (FN) for a dataset that included Dixi SEEG. Electrode locations relative to the brain surface (left) and CT image (right) are shown. **(C)** Histogram of optimal thresholds in Hounsfield units (HU) for all 48 datasets demonstrates a bimodal distribution with two peaks at 2200 and 3400 HU. **(D)** Examples showing the number of detected electrodes as a function of threshold for two datasets with both a low (left) and a high (right) optimal threshold (red circle). The overall median threshold (2365 HU) for all 48 datasets is indicated with a red vertical line.

Automatic electrode detection has been included in LeGUI. The detection algorithm was designed to be robust across datasets and electrode types. This is achieved by searching for an optimal threshold that is unique to each dataset based on stability in the detection rate or sensitivity (see Materials and Methods). This algorithm was tested on 48 datasets (4695 electrode contacts) and demonstrated good performance, with an overall sensitivity of 0.93 and only 71 false positives. Results were grouped by electrode type (ECoG vs. Ad-Tech SEEG vs. Dixi SEEG) (Figure 3A), and the detection performance was highest (pairwise rank-sum, *p* < 0.01) for Ad-Tech SEEG, with a median rate and range of 1 (0.94–1). Ad-Tech SEEG also had the lowest number of false-positive detections, with a median of 0 (0–7). Ad-Tech ECoG and Dixi SEEG had lower detection performance, with rates of 0.87 (0.75–0.99) and 0.87 (0.65–1), respectively, and higher false positives of 2.5 (0–18) and 1 (0–3), respectively. The lower performance for these electrode types is likely due to the smaller electrode contact size and intercontact spacing of the Dixi electrodes and the more complex and overlapping implant geometry of ECoG grids and strips. Dixi SEEG leads oriented perpendicular to the CT slice plane typically showed inferior performance and more false positives (Figure 3B). The small intercontact spacing (3.5 mm) of the Dixi leads and the lower resolution of the CT along the slice plane likely contributed to this outcome.

Because the detection algorithm works by finding an optimal threshold for each dataset, optimal thresholds for all 48 datasets were analyzed. The median optimal threshold and range was 2365 (1789–3977) Hounsfield units (HU). Optimal thresholds also demonstrated a bimodal distribution across datasets, with two distinct peaks occurring at approximately 2200 and 3400 HU (Figure 3C). When grouped by electrode type, no significant difference in threshold was found [Kruskal-Wallis (KW) test, df = 2, χ^2^ = 2.47, *p* = 0.29], indicating that the bimodality of optimal thresholds could not be attributed to the type of electrode implanted. These results indicate the potential benefit of using multiple thresholds when detecting electrodes across different datasets. Examples are given for two datasets that have a low (Figure 3D, left) and a high (Figure 3D, right) optimal threshold, respectively. The number of detected electrodes as a function of threshold are shown for each dataset. The median optimal threshold from all 48 datasets is shown as a vertical red line. It is clear from these two examples that prespecifying a threshold would diminish the automatic detection performance.

### Electrode Alignment and Projection

**FIGURE 4:**
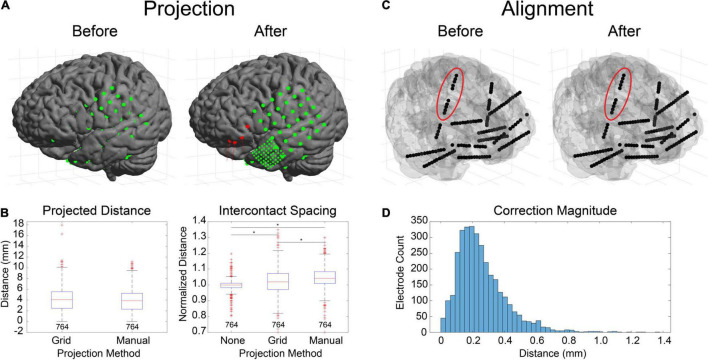
Electrode projection and alignment results. **(A)** Example showing electrodes before (left) and after (right) projection using the “manual” projection method. An 8×8 ECoG grid and multiple strips (10-mm spacing) along with an 8×8 mini-ECoG grid (3-mm spacing) are visible. Projection vectors (red lines) are shown for 4 selected electrode contacts (red spheres) that represent the normal vector to the “projection” surface (not shown). **(B)** Projection distances for 10 datasets (764 electrodes) for the “grid” (Hermes et al., 2010) and “manual” projection methods are shown (left). The median and ranges were 4.07 (0.00–17.95) and 3.88 (0.00–11.17) mm, respectively. No differences were found between the two methods (KW test, df = 1, χ^2^ = 3.14, *p* = 0.076). Intercontact spacings were measured for unprojected electrode contacts, as well as for the two projection methods (right). The median and ranges were found to be 1.00 (0.45–1.78), 1.02 (0.38–1.93), and 1.04 (0.44–1.99) mm, respectively. Asterisks indicate significance (pairwise rank sum, *p* < 0.01). Separations for the “grid” and “manual” methods were both higher than unprojected “none,” indicating expansion during the projection process. **(C)** Example showing electrodes before (left) and after (right) alignment process. Corrections to electrode positions were small but can be visualized in the right insular lead (red ellipse). **(D)** Correction magnitudes for 38 datasets (365 leads, 3726 contacts). The median and range were 0.23 (0.00–1.37). The median correction magnitude of 0.23 mm was less than the voxel resolution (0.4 mm/vox), suggesting that most corrections are for rounding errors due to the voxelization of the image.

There are several ways in LeGUI to adjust the positions of electrodes to correct for brain shift or inaccurate centroid calculations due to variable-quality CT scans or rounding errors when converting between voxels and millimeters. Brain shift correction or electrode projection to the smoothed brain (projection) surface for ECoG grids or strips can be performed using a fully automated routine where the projected direction is the local normal to the electrode (grid) surface (Hermes et al., 2010). Projection can also be performed manually for selected electrodes where the projected direction is the local normal of a nearby patch of the projection surface. The advantage of this method is that it does not require prior label assignments to be made. Both methods were tested using 10 ECoG datasets containing 764 electrode contacts (Figures 4A,B). An example is shown of the projection results using the “manual” method for a dataset with a standard grid (Ad-Tech FG64C-SP10X-000, 10-mm spacing) and a mini grid with smaller electrode contacts and closer spacing (Ad-Tech FG64C-MP03X-000, 3-mm spacing) (Figure 4A). Projected distances (median and range) across all datasets for the “grid” approach were 4.07 (0.00–17.95) mm. Projected distances for the “manual” approach were 3.88 (0.00–11.17) mm. No significant difference was found between these two approaches (KW test, df = 1, χ^2^ = 3.14, *p* = 0.076) (Figure 4B, left). Intercontact distances were also measured before and after projection to quantify possible expansion or contraction. Distances were normalized by dividing by the known intercontact spacing for a given grid or strip. The median and range of unprojected normalized intercontact distances for all 10 datasets were 1.00 (0.45–1.78) mm. After “grid” projection, the normalized distances were higher than the unprojected at 1.02 (0.38–1.93) mm. After “manual” projection, the distances were higher than the “grid” projected distances at 1.04 (0.44–1.99) mm (Figure 4B, right). These results show that projection using either of the two methods results in a slight expansion of the intercontact distances; however, the “manual” projection method showed a slightly higher expansion rate.

Electrode alignment is a method that is primarily used to “straighten” and regularize the intercontact spacing of SEEG electrodes as a result of inaccurate centroid calculations from variable-quality CT scans or voxel-related rounding errors. An example of this is shown for a dataset with 12 bilateral SEEG leads (Dixi Medical Microdeep) (Figure 4C). Correction magnitudes are small and difficult to visualize. However, they are most noticeable for the vertically oriented right insula lead (Figure 4C, red ellipse). Correction magnitudes (median and range) measured for 38 datasets containing 365 SEEG leads (3726 electrode contacts) were 0.23 (0.00–1.37) mm (Figure 4D). Normalized intercontact spacings were also measured before and after correction and found to be 1.00 (0.70–1.24) and 1.00 (1.00–1.00).

### Anatomical Localization

**FIGURE 5:**
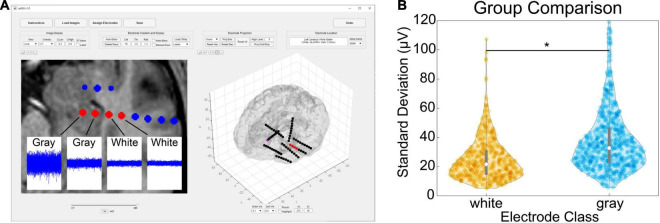
Comparison of gray and white segmentation results using electrophysiology recordings. **(A)** Patient-specific example showing SEEG electrodes (AdTech BF08R-SP05X-000, 5-mm spacing) in left hippocampus transitioning to white matter (left to right) with corresponding LFP data (1-kHz sampling rate, 0.3-Hz high-pass filter). Each data plot spans 1.5 s along the x-axis and 1.5 mV along the y-axis and displays 415 overlaid time segments distributed throughout an approximately 50-min recording session. Standard deviations from left to right were 95 (gray), 38 (gray), 23 (white), and 21 (white) μV, respectively. **(B)** Comparison of recording amplitude (standard deviation) from 17 patients for a total of 1066 gray/white classified electrode contacts (456 white, 610 gray). Gray electrodes recorded higher amplitudes compared with white (pairwise rank sum, *p* < 0.001). Asterisk indicates significance.

Verification of the accuracy of anatomical localization in LeGUI was performed by comparing features of the recorded electrophysiology data with electrode location. Local field potential (LFP) power in the lower frequencies (<150 Hz) has been shown to be a good predictor of electrode location (gray or white matter) (Greene et al., 2021). Instead of power, we used the standard deviation of resting-state LFP (1-kHz sampling rate, 0.3-Hz high-pass filter) as a feature to compare electrodes in both gray and white matter for 17 datasets (Figure 5). An example of the LFP recordings for several electrodes that traverse a gray and white matter boundary is shown for patient 31 (Figure 5A, insets). Multiple 1.5-s segments of LFP data are shown overlaid for each electrode. The amplitude of the LFP signal decreases in the left-to-right direction as the electrodes move from a region of gray matter to a region of white. A group comparison was also performed between LFP amplitude (standard deviation) for gray and white matter electrodes for 17 datasets (1066 total electrode contacts, 456 white, 610 gray) (Figure 5B). Gray matter electrodes had higher LFP amplitudes with a median and range of 33.4 (5.6–248.7) μV than white matter electrodes with a median and range of 21.6 (5.1–186.3) μV (KW test, df = 1, χ^2^ = 139.9, *p* = 2.8e-32).

**FIGURE 6:**
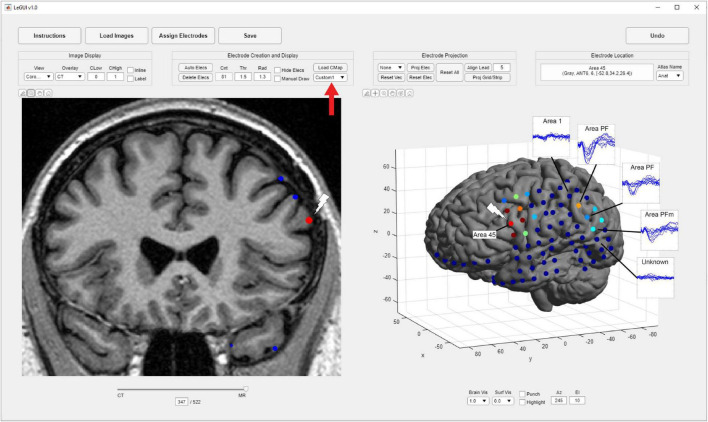
Corticocortical evoked potentials (CCEPs) demonstrate a connection between the inferior frontal lobe (IFL) and the inferior parietal lobule (IPL). Single-pulse stimulation was applied to an ECoG electrode in area 45 of IFL (white lightning bolts), and CCEPs were recorded in areas PF and PFm of IPL (blue traces) demonstrating functional connectivity between these areas. These traces are overlaid for the purposes of this figure and are not a feature of LeGUI. Locations were determined using a cytoarchitectural atlas (SPM Anatomy toolbox) by selecting electrodes and viewing the corresponding anatomical locations in LeGUI (upper right). A custom colormap was used to color electrodes based on CCEP size (red = largest, blue = smallest). The colormap was loaded using a dropdown menu provided in LeGUI (red arrow).

In addition to gray and white matter comparisons, anatomical localization was demonstrated in a single patient by comparing the propagation of corticocortical evoked potentials (CCEPs) during single-pulse stimulation with the anatomical labels of the SPM Anatomy toolbox atlas as displayed in LeGUI (Figure 6). Single-pulse stimulation (amplitude = 7.5 mA, pulse width = 0.5 ms) was applied to an ECoG electrode in a 32-channel grid (Ad-Tech FG32C-SP10X-000) over Brodmann area 45 in the left inferior frontal lobe (IFL). CCEPs were measured on the remaining 31 electrodes of that grid, as well as 32 electrodes of another grid placed over the inferior parietal lobe (IPL). The CCEP data was sampled at 1 kHz and filtered using a 0.3–250 Hz Butterworth band-pass filter. Electrodes in PF and PFm of IPL recorded CCEPs following stimulation in IFL indicating a functional connection between the site of stimulation and these areas. Structural connectivity between these areas has been previously demonstrated with probabilistic tractography (Caspers et al., 2011).

**FIGURE 7:**
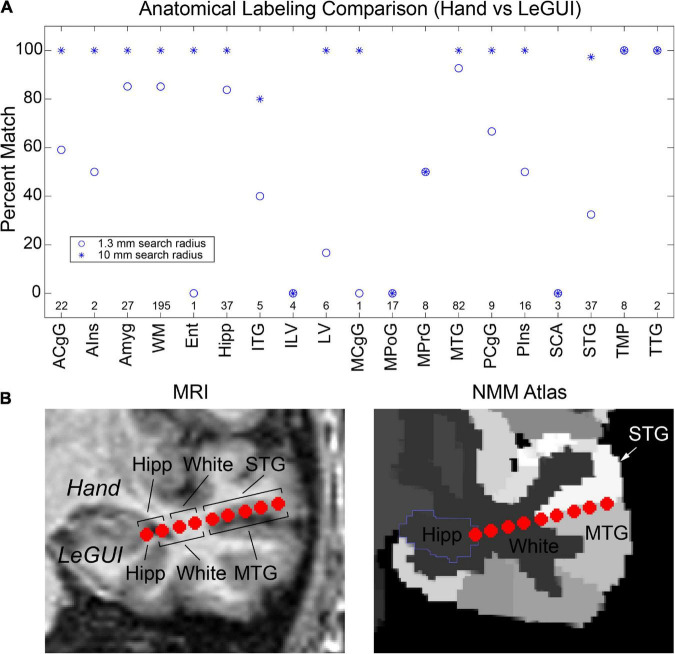
Anatomical electrode labels generated by LeGUI closely match electrodes labeled by hand by an experienced neuroanatomist (K.W.). **(A)** Percent match of LeGUI labels with hand labels grouped by NMM label type. Left and right hemisphere labeling was removed for clarity. Circles indicate percent match for LeGUI labels generated using a 1.3-mm radius sphere around each electrode. Asterisks indicate percent match for labels generated using a 1-cm sphere (see Materials and Methods). Numbers above each label indicate count. **(B)** Example of SEEG electrode labels for a lead placed into the left temporal lobe. Electrodes are shown relative to the MRI (LeGUI inline projection feature) with the hand labels indicated above and LeGUI labels below (left). Electrodes are also shown relative to the NMM atlas (right). A mismatch can be seen between the hand (STG) and LeGUI (MTG) labels for the 5 lateral contacts. These electrodes closely follow the border between STG and MTG in the NMM atlas (right). ACgG, anterior cingulate gyrus; AIns, anterior insula; Amyg, amygdala; WM, white matter; Ent, entorhinal area; Hipp, hippocampus; ITG, inferior temporal gyrus; ILV, inferior lateral ventricle; LV, lateral ventricle; MCgG, middle cingulate gyrus; MPoG, medial postcentral gyrus; MPrG, medial precentral gyrus; MTG, middle temporal gyrus; PCgG posterior cingulate gyrus; PIns, posterior insula; SCA, subcallosal area; STG, superior temporal gyrus; TMP, temporal pole; TTG, transverse temporal gyrus.

Finally, verification was further performed by comparing hand labeling of six datasets (482 electrode contacts) from two different institutions (University of Utah and University of Washington) with the anatomical labels produced by LeGUI using the NMM atlas. Hand labeling was performed by an experienced neuroanatomist (K.W.). Out of the 482 electrode contacts, labels from 353 electrode contacts (73%) assigned by LeGUI matched the hand labels. These LeGUI labels were based on the most common voxel type contained within a 1.3-mm radius sphere surrounding the electrode center (see Materials and Methods). When grouped by region, LeGUI showed high percentage matches (>80%) for amygdala, white matter, hippocampus, and middle temporal gyrus (Figure 7A, circles). LeGUI did not perform as well (<40%, >5 data points) for lateral ventricle, medial postcentral gyrus, and superior temporal gyrus. An example of the mismatch between hand and LeGUI labels for five lateral temporal contacts is shown in Figure 7B.

Importantly, in addition to the 1.3-mm radius labels, LeGUI also generates a list of labels contained within a 1-cm sphere around each electrode sorted by percent volume from highest to lowest (see Materials and Methods). Using these labels (excluding volumes <5% of the sphere), LeGUI matched 452 out of 482 hand labels (94%). When grouped by region, most labels showed a 100% match (Figure 7A, asterisks).

## Discussion

Locate electrodes Graphical User Interface was designed to be a fast and accurate tool for detecting and anatomically localizing intracranial electrodes in patients undergoing epilepsy monitoring. Its versatility also allows for localization of multiple types of chronically implanted intracranial electrodes, including those used for deep brain stimulation (DBS) and responsive neurostimulation (RNS). LeGUI has minimal dependencies and few installation steps. Installation includes downloading the compiled software executable for Windows, Mac, or Linux platforms and downloading and installing the freely available MATLAB runtime environment. It requires minimal knowledge of medical imaging and has a short learning curve, making it a useful tool for researchers with varying skillsets.

Though LeGUI has not been evaluated by regulatory bodies (e.g., FDA), several features of LeGUI were designed for clinician researchers. The initial image-processing, coregistration, and segmentation steps are fast (∼30 min) relative to other software packages (Blenkmann et al., 2017; Groppe et al., 2017; Qin et al., 2017), making it suitable for the typical clinical workflow. For a clinician, it is important to be able precisely localize electrodes to understand how they interface with the nervous system. One of the best ways to localize electrodes is to visualize their positions relative to the anatomical imaging. Therefore, we emphasized visualizations of the coregistered CT and MRI images in LeGUI. The unique interactive split-screen design showing both 2D and 3D representations of electrodes relative to the brain makes navigating and visualizing electrodes intuitive and efficient. Like other clinical software packages (Gumprecht et al., 1999), both the MRI and the CT images can be viewed in 2D, with the ability to select sagittal/coronal/axial viewing planes as well as scroll through slices using the keyboard or mouse. Brightness and contrast (window-level and width) can also be adjusted with a mouse drag over the image in both the vertical and the horizontal directions, respectively. A slider at the bottom of the image fades the MRI with respect to the CT, allowing for the visualization of the accuracy of the coregistration step as well as the ability to compare electrode location in the CT relative to the MRI. Once electrodes have been detected, the 2D plot also displays each electrode location embedded within the MRI for easy visual inspection relative to MRI features.

After electrodes have been labeled, a SEEG lead can be rotated to match the viewing plane (inline projection) so that all electrode contacts within a lead can be viewed in the same slice plane and easily visualized relative to surrounding MRI structures. Information about selected electrodes, such as location relative to the active atlas, gray/white classification, label, and channel number, is displayed on the screen.

Locate electrodes Graphical User Interface comes bundled with two atlases, the NMM atlas and the SPM Anatomy toolbox atlas. These atlases provide labels for both broad and more specific regions of the brain, with specificity down to the level of Brodmann areas. LeGUI also comes with the ability to load custom MNI-registered atlases for users that need this expanded capability.

Locate electrodes Graphical User Interface has several features targeted to researchers and more advanced users. These features include an intuitive and efficient interface for labeling electrodes and assigning data channels for analysis, automatic electrode detection, automatic electrode projection to the brain surface for ECoG grids and strips, automatic electrode alignment for electrode leads (SEEG, DBS, RNS), and the ability to manually manipulate electrode location if the automated routines fail. In addition, all data relating to the electrodes is saved to a MATLAB (.mat) file for later use in analysis. This file includes electrode location in patient space, electrode location in MNI space, electrode location relative to each of the loaded atlases, and electrode class (gray or white). All saved electrode data are sorted by channel number, simplifying the link between localized electrodes and the analysis of electrophysiological data. Many groups still rely on manual techniques to anatomically label electrodes (Sato et al., 2011; Lega et al., 2015; Zhao and Wang, 2018; Jiang et al., 2019; Zheng et al., 2019) or use different software packages for each of the localization steps (Azarion et al., 2014; Blenkmann et al., 2017; Groppe et al., 2017; Laplante et al., 2017), which can lead to inconsistent results. Because LeGUI contains all the steps needed for localization in a single interface, it can help researchers establish a standardized pipeline that produces consistent results and boosts overall productivity.

Other software packages incorporate some of the same features as LeGUI, such as image coregistration and normalization, automatic electrode detection, electrode projection, anatomical labeling, and 2D and 3D visualizations of electrodes relative to the imaging. However, to our knowledge, none of the available packages include all these features bundled together in a single multifunctional interface that is available across multiple operating systems. A comparison of features of LeGUI with several other intracranial electrode localization packages is provided in Table 1.

**TABLE 1:** Comparison of intracranial electrode localization packages.

Name	URL	Features	Testing/Performance	Platform/Dependencies
LeGUI	https://github.com/rolston-lab/legui	– MR/CT coregistration – MR normalization – Automatic electrode detection – Brain shift correction – Electrode labeling – Anatomical localization – Custom atlases – SEEG/ECoG compatibility – 2D/3D visualizations – Anatomical overlays – Single unified GUI	– Tested on 51 datasets (5089 electrode contacts) – ∼30 min user time, ∼30 min computer time – Automatic electrode detection showed 93% true positive rate – Median brain shift correction (3.88 mm, *n* = 764, ECoG only) – Median SEEG correction (0.23 mm, *n* = 3726) – Anatomical labels showed 73% (1.3-mm radius) or 94% (1-cm radius) match with hand labels by expert – Gray/white classifications verified using electrophysiology recordings – Superior longitudinal fasciculus confirmed using anatomical labels and cortico-cortical evoked potentials	– Windows, Mac, Linux – MATLAB Runtime (executables), MATLAB (source code), SPM (bundled with software)
iElvis	https://github.com/epiSurg/EpiSurg	– MR/CT coregistration – MR normalization – Manual electrode detection – Brain shift correction – Anatomical localization – ECoG compatibility – 2D/3D visualizations – Anatomical overlays	– Tested on 5–8 datasets – 30–60 min user time, 12–24 h computer time – Brain shift correction accuracy measured using interoperative photographs (3-mm error, Dykstra algorithm) or (0.74-mm error, Yang algorithm) – Hand sensorimotor cortex confirmed using fMRI, iEEG, and iEBS	– Mac, Linux – MATLAB, FreeSurfer, BioImage, FSL
ALICE	https://github.com/UMCU-RIBS/ALICE	– MR/CT coregistration – Automatic electrode detection – Electrode labeling – Brain shift correction – ECoG and high-density ECoG – 2D/3D visualizations	– Tested on 17 datasets – 30–60 min total processing time – Locations compared to previous algorithm (Hermes et al., 2010) showing 1.66-mm error – High-density locations compared to intraoperative photographs showing 1.01- and 1.94-mm error for 2 datasets, respectively	– Mac, Linux – MATLAB, AFNI, Suma, FreeSurfer
iElectrodes	https://sourceforge.net/projects/ielectrodes	– MR/CT coregistration – MR normalization – Semi-automatic detection – Automatic electrode labeling – Brain shift correction – Anatomical localization – SEEG/ECoG compatibility – 2D/3D visualizations	– Tested on 22 datasets (1242 electrode contacts) – 2–3 min per electrode array user time, ∼7 h computer time – Locations compared to expert manual evaluators with 0.56-mm error (5 datasets, 91 electrode contacts)	– Mac, Linux – MATLAB, SPM, FreeSurfer, FSL
iELU	https://github.com/aestrivex/ielu	– MR/CT coregistration – Automatic electrode detection – Automatic electrode labeling – Brain shift correction – SEEG/ECoG compatibility – 2D/3D visualizations	– Tested on 12 datasets – ∼30 min total processing time Automatic electrode detection/sorting 94.8% true positive rate (ECoG only)	– Mac, Linux – Python, chaco, matplotlib, Mayavi, MNE-python, nibabel, PySurfer, PyMCubes, FreeSurfer
iEEGView	https://github.com/GuangyeLiGit/iEEGview.git	– MR/CT coregistration – MR normalization to MNI and FreeSurfer standard spaces – Semi-automatic detection – Brain shift correction (ECoG + SEEG) – Electrode labeling – Anatomical localization – SEEG/ECoG compatibility – 2D/3D visualizations	– Tested on 28 datasets (3756 electrode contacts) – 30–60 min user time, 8–24 h computer time – Average brain shift correction for ECoG (3.71 mm, *n* = 568) and SEEG (0.89 mm, *n* = 32)	– Mac only – MATLAB, FreeSurfer

Most packages provide coregistration of the CT and MRI, normalization of the MRI to a standardized space, and segmentation of the different MRI tissue planes. These operations are typically performed using FreeSurfer^5^ (Dykstra et al., 2012; Gramfort et al., 2013; Blenkmann et al., 2017; Groppe et al., 2017; Laplante et al., 2017; Narizzano et al., 2017; Qin et al., 2017; Branco et al., 2018; Li et al., 2019), an open-source software suite that is available for Linux and Mac environments but not Windows. However, FreeSurfer is often run from the command line, making it difficult to install and use for the novice user. Additionally, although FreeSurfer can perform the normalization and provide detailed segmentations of the MRI, including segmentation of subcortical structures, these processes can take up to 24 h to complete (Groppe et al., 2017). In contrast, the image-processing steps in LeGUI are done using SPM, which is written in the MATLAB programming language and take approximately 30 min to complete. Because all operations are performed in the background and are controlled using a simple user interface, even novice users can process a dataset. Further, standalone executables are provided for LeGUI, so users without an active MATLAB license can still run the program. As part of the image-processing steps, LeGUI also provides the option to align the images to ACPC space. ACPC alignment can be performed with FreeSurfer; however, this is typically not incorporated as an option into most packages.

Automatic electrode detection has the potential to significantly decrease user time if the detection sensitivity and accuracy are high. Some packages include automatic detection as a feature (Laplante et al., 2017; Qin et al., 2017; Branco et al., 2018); however, some of these packages can detect only ECoG surface or SEEG depth electrodes, but not both. Several groups have developed new methods or algorithms to improve detection sensitivities and accuracies (Ekstrom et al., 2008; Granados et al., 2018; Hinds et al., 2018; Centracchio et al., 2021), but these algorithms are standalone and have yet to be included in a full-featured software package.

Locate electrodes Graphical User Interface includes an algorithm for automatically detecting electrodes that performs well for electrodes of different types, sizes, and spacings. This algorithm works by searching for the optimal threshold unique to each dataset. Detection performance was found to be similar to other automated algorithms (Laplante et al., 2017). Performance was better for Ad-Tech SEEG electrodes than for ECoG grids and strips and Dixi SEEG electrodes. The difference for grids and strips is likely due to the frequent overlap and more complex geometry of these implants. Several of our datasets contained a grid and multiple overlapping strips, making it difficult to distinguish between the individual overlapping electrodes or contacts. We also had a dataset where a grid and strips were passed into the interhemispheric fissure on both the right and left side of the brain, resulting in a similar overlap condition (P5). Grids and strips were also found to have interconnecting wires that produced more prominent artifact in the CT, resulting in false detections. The decreased performance for Dixi SEEG is likely due to the smaller contact size (0.8-mm vs. 0.86-mm diameter; 2-mm vs. 2.29-mm length) and spacing (3.5 mm vs. 5 mm). Detection sensitivity was also lower for leads placed perpendicular to the slice plane (i.e., superior/inferior leads for an axial-sliced image), a dimension that typically has a lower resolution (1 mm/vox vs. <0.5 mm/vox). We found that a slice resolution of 0.6 mm and a bone window setting worked best to detect these electrodes. We also found that detection performance along the slice dimension for Dixi electrodes could be improved by increasing the detection threshold. However, increasing this threshold resulted in more false-negative detections and an overall decrease in the detection sensitivity, suggesting the need for multiple thresholds within a single dataset. In general, we found that optimal thresholds for all 48 datasets produced a bimodal distribution with peaks that differed by approximately 1000 HU. This suggests that a single threshold applied to any arbitrary dataset has nearly a 1 in 2 chance of producing suboptimal detection results. Future improvements to the detection algorithm might include the use of multiple thresholds within a single dataset, as well as prior knowledge of the geometry of the implanted electrodes to further improve the sensitivity and minimize false electrode detections.

Many packages include the option to project ECoG electrodes to the preimplant brain surface to account for brain shift from surgery (Blenkmann et al., 2017; Groppe et al., 2017; Hamilton et al., 2017; Laplante et al., 2017; Qin et al., 2017; Branco et al., 2018; Li et al., 2019). There are several algorithms that are used to accomplish this task that vary from projection along a local normal vector to the electrode (grid) surface (Hermes et al., 2010) to energy-minimizing algorithms that seek to maintain intercontact spacing during the projection process (Dykstra et al., 2012) or algorithms that use a surface-based grid of regions of interest to guide the projection (Trotta et al., 2018). LeGUI provides electrode projection using a well-known algorithm that projects in the direction of the local normal vector to the electrode (grid) surface (Hermes et al., 2010). It also includes the option to manually project if the automated method fails. Projection distances were found to be similar to those reported in the literature (Li et al., 2019). However, the intercontact spacing was not strictly preserved during both automated and manual projection, leading to a slightly expanded representation of a grid or strip. This was more pronounced if the grid or strip contained regions of large curvature (i.e., partially inserted into a sulcus) or was being projected onto a region of the surface with large curvature. Because these errors can be easily visualized in LeGUI, corrections can be made using the manual features on an electrode-by-electrode basis. Future improvements might include incorporating more advanced algorithms for projecting electrodes that seek to preserve intercontact spacing (Dykstra et al., 2012).

Additionally, LeGUI has a feature to correct intercontact spacing and alignment of SEEG leads based on the known geometry of the lead. Errors in spacing can arise from poor-quality CT scans or voxel-related rounding errors during the detection process. Correcting lead geometry is important when creating models of the electrodes and surrounding brain tissues for estimating activation volumes during stimulation or for signal source localization during recording. This type of correction performed well on the 38 SEEG datasets that were tested, showing a median correction magnitude of 0.23 mm (Figures 4C,D). This amount of correction is approximately half of the image resolution (0.4 mm/voxel), suggesting that most of the correction is accounting for rounding errors due to the voxelization of the images. A few failure modes were observed for the correction process across these datasets. Correction was observed to fail when the first electrode/contact of an SEEG lead was not aligned well with the corresponding CT artifact. This behavior is expected because the algorithm begins at the first contact and assumes that it is properly aligned with the CT. Correction also failed if the SEEG lead contained a large bend. For some leads, the correction process was observed to slightly expand or contract the electrodes, leading to a mismatch with the CT image. This happened infrequently and is likely a result of compounding correction errors as the algorithm moves up the contacts in a lead because of a slight mismatch between the known intercontact spacing and the actual spacing within the CT image. Future improvements to this algorithm might include fitting electrodes to functions with some curvature to account for bends in a lead and remove the dependency on the first electrode similar to the approach taken by another group (Granados et al., 2018). The side-by-side display of 2D and 3D images in LeGUI facilitates comparison of reconstructed electrodes with the raw images, aiding in the user’s recognition of any alignment and projection errors.

Anatomical localization of electrodes using brain atlases can provide important information about the location of electrodes relative to surrounding brain structures. This information can be used to estimate the source of signals that are recorded on the electrodes or to predict the brain regions that might be activated when stimulation is applied to those electrodes. Most packages include anatomical localization using one or more bundled atlases and provide some type of anatomical overlay so electrodes locations can be visualized relative to the different regions of each atlas (Blenkmann et al., 2017; Groppe et al., 2017; Li et al., 2019). However, some packages do not provide this feature, requiring the user to perform the normalization step separately and transform the electrode coordinates to this standardized space on their own (Laplante et al., 2017; Branco et al., 2018). Further, most packages come bundled with a fixed set of atlases and do not provide the option of importing new custom atlases.

Locate electrodes Graphical User Interface provides anatomical localization of electrodes for two atlases that come bundled with the software (NMM and SPM Anatomy toolbox). In addition, LeGUI allows the user to import any MNI-registered atlas that might be of interest. This is a valuable feature for researchers who are studying specific regions of the brain and need more detailed maps of those areas. LeGUI also provides a rich set of visualizations including overlays of each of the imported atlases and the ability to rotate the images so electrodes can be viewed in a single 2D slice plane (inline projection), further enhancing the user’s ability to reconstruct electrode positions within a complex 3D space.

Anatomical localization of the electrodes in LeGUI is performed by finding the most common labeled voxel within an electrode sphere for the selected MNI-registered atlas. Voxels are warped from patient space to atlas space using the deformation fields that are generated by SPM during the normalization step. Validation was performed by comparing anatomical electrode labels from LeGUI to labels that were manually assigned by an experienced neuroanatomist. This comparison demonstrates that the automatic labels from LeGUI are similar to hand labeling by an expert, representing a significant savings in time and labor. Overall, LeGUI performed well, with a high percentage match to the manual labels for most brain regions. However, performance was lower for some brain regions, such as the medial postcentral gyrus and superior temporal gyrus (Figure 7A). Potential methodological discrepancies or partial volume effects between the two labeling approaches may account for these differences. Performance improved when the probabilistic atlas labels generated by LeGUI were used to find a match. These labels contain a list of volumes occupied by nearby anatomical regions within a 1-cm radius sphere around each electrode. The 1-cm radius was chosen to provide a conservative search volume for estimating potential sources of signals recorded on an electrode. Low frequency signals can have sources as distant as 1 to 2 cm from an electrode (Muller et al., 2016). The hand labeling was primarily limited to temporal lobe and mid-line limbic structures due to the vast gyral complexity of human prefrontal and parietal association cortices in 2D. While limited in scope, the hand labeling shows that the SPM normalization step was successful and that the non-linear transforms from patient to MNI space in LeGUI have been properly implemented. Additional labeling using locations outside these areas would only further validate SPM, an already well-established and highly cited software package for medical image processing. However, because labels outside these areas were not formally evaluated, the level of uncertainty is unknown. Validation was also performed by comparing evoked potentials during single-pulse electrical stimulation with corresponding labels from LeGUI for a single dataset (P15) (Figure 6). This comparison demonstrated a functional connection between the inferior frontal lobe (Brodmann area 45) and the inferior parietal lobule (PF and PFm), which is a known connection (superior longitudinal fasciculus) that has been validated using structural connectivity metrics (Caspers et al., 2011; Barbeau et al., 2020). LeGUI assigns a gray or white classification to electrodes based on the most common voxel type within the electrode sphere taken from the SPM gray and white segmented images. This was validated by showing that electrodes classified as white matter exhibited lower-amplitude electrophysiological recordings when compared with electrodes classified as gray matter (Figure 5). These results all support the accuracy of electrode localization using LeGUI.

Locate electrodes Graphical User Interface is a modular software program. As new or more efficient methods of electrode localization, anatomical localization, and visualization are created, LeGUI can incorporate these methods as optional or standard algorithms. This modularity is already present in the software’s ability to dynamically add atlases and remap electrode labels. As usability was a major concern in the development of LeGUI, feedback has been actively solicited internally and across institutions. Feedback from users has already led to significant improvements in layout, workflow, and feature availability. To encourage a larger base of users and continue to improve LeGUI, additional resources like video and written tutorials and local workshops will be planned.

## Conclusion

Locate electrodes Graphical User Interface has been optimized for speed and ease of use, making it suitable for a wide range of users with varying levels of experience in medical imaging and image processing. The visualizations and user interactions that are incorporated into LeGUI allow for a seamless and intuitive exploration of the image space, resulting in efficient localization of electrodes. Many automated routines have been included to further optimize the task of localizing electrodes, such as automatic electrode detection, ECoG electrode projection to correct for brain shift, and SEEG lead alignment. If the automated routines fail, the ability to manually detect and reposition electrodes has been included as an alternative. Finally, the location of electrodes relative to common MNI-based atlases and the ability to load custom atlases has been included. All of these features combined together into a single interface make LeGUI a valuable tool for both clinicians and researchers.

## Data Availability Statement

The datasets presented in this article are not readily available because they are protected by the Health Insurance Portability and Accountability Act (HIPAA). Requests are subject to the establishment of a data sharing agreement between the University of Utah and the requesting institution or individual. Requests to access the datasets should be directed to TD, tyler.davis@hsc.utah.edu

## Ethics Statement

The studies involving human participants were reviewed and approved by the University of Utah Institutional Review Board. Written informed consent for participation was not required for this study in accordance with the national legislation and the institutional requirements.

## Author Contributions

TD developed the software and wrote the manuscript. KW hand labeled datasets for validating the software and wrote sections of the manuscript. All authors contributed to the design and testing of the software and manuscript revision, read, and approved the submitted version.

## Conflict of Interest

The authors declare that the research was conducted in the absence of any commercial or financial relationships that could be construed as a potential conflict of interest.

## Publisher’s Note

All claims expressed in this article are solely those of the authors and do not necessarily represent those of their affiliated organizations, or those of the publisher, the editors and the reviewers. Any product that may be evaluated in this article, or claim that may be made by its manufacturer, is not guaranteed or endorsed by the publisher.

## Footnotes

1
https://www.fil.ion.ucl.ac.uk/spm/software

2
https://nifti.nimh.nih.gov

3
http://Neuromorphometrics.com

4
https://fsl.fmrib.ox.ac.uk/fsl/fslwiki/FSL

5
www.freesurfer.net
