# Corrigendum: LeGUI: A Fast and Accurate Graphical User Interface for Automated Detection and Anatomical Localization of Intracranial Electrodes

**DOI:** 10.3389/fnins.2022.858978

**Published:** 2022-02-16

**Authors:** Tyler S. Davis, Rose M. Caston, Brian Philip, Chantel M. Charlebois, Daria Nesterovich Anderson, Kurt E. Weaver, Elliot H. Smith, John D. Rolston

**Affiliations:** ^1^Department of Neurosurgery, University of Utah, Salt Lake City, UT, United States; ^2^Department of Biomedical Engineering, University of Utah, Salt Lake City, UT, United States; ^3^Department of Pharmacology and Toxicology, University of Utah, Salt Lake City, UT, United States; ^4^Department of Radiology, University of Washington, Seattle, WA, United States; ^5^Department of Biological Structure, University of Washington, Seattle, WA, United States

**Keywords:** MATLAB, anatomical localization, graphical user interface (GUI), electrocorticography (ECoG), software, stereotactic electroencephalography (SEEG), intracranial electrode localization

In the original article, the reference for Antonio Valdes-Sosa et al., 2017 was incorrectly written as “Antonio Valdes-Sosa, P., Nagarajan, S. S., Arnulfo, G., Horn, A., Blenkmann, A. O., Phillips, H. N., et al. (2017). iElectrodes: a comprehensive open-source toolbox for depth and subdural grid electrode localization. *Front. Neuroinform*. 11:14. 10.3389/fninf.2017.00014.” It should instead be written as “Blenkmann, A. O., Phillips, H. N., Princich, J. P., Rowe, J. B., Bekinschtein, T. A., Muravchik, C. H., et al. (2017). iElectrodes: a comprehensive open-source toolbox for depth and subdural grid electrode localization. *Front. Neuroinform*. 11:14. 10.3389/fninf.2017.00014,” with “Blenkmann et al., 2017” as the in-text citation.

Additionally, the reference for Jiang T. et al., 2017 was incorrectly written as “Jiang, T., Wang, J., Lin, J. J., Wang, L., Qin, C., Tan, Z., et al. (2017). Automatic and precise localization and cortical labeling of subdural and depth intracranial electrodes. *Front. Neuroinform*. 11:10. 10.3389/fninf.2017.00010.” It should be “Qin, C., Tan, Z., Pan, Y., Li, Y., Wang, L., Ren, L., et al. (2017). Automatic and precise localization and cortical labeling of subdural and depth intracranial electrodes. *Front. Neuroinform*. 11:10. 10.3389/fninf.2017.00010,” with “Qin et al., 2017” as the in-text citation.

The authors apologize for these errors and state that they do not change the scientific conclusions of the article in any way. The original article has been updated.

## Publisher's Note

All claims expressed in this article are solely those of the authors and do not necessarily represent those of their affiliated organizations, or those of the publisher, the editors and the reviewers. Any product that may be evaluated in this article, or claim that may be made by its manufacturer, is not guaranteed or endorsed by the publisher.

## References

[B1] Blenkmann A. O. Phillips H. N. Princich J. P. Rowe J. B. Bekinschtein T. A. Muravchik C. H.. (2017). iElectrodes: a comprehensive open-source toolbox for depth and subdural grid electrode localization. Front. Neuroinform. 11:14. 10.3389/fninf.2017.00014 28303098 PMC5333374

[B2] Qin C. Tan Z. Pan Y. Li Y. Wang L. Ren L.. (2017). Automatic and precise localization and cortical labeling of subdural and depth intracranial electrodes. Front. Neuroinform. 11:10. 10.3389/fninf.2017.00010 28261083 PMC5314105

