# Supplementary figures and images for: LeGUI: A Fast and Accurate Graphical User Interface for Automated Detection and Anatomical Localization of Intracranial Electrodes
Source: Front Neurosci. 2021 Dec 9;15:769872. doi: 10.3389/fnins.2021.769872 (PMC8695687; doi:10.3389/fnins.2021.769872)

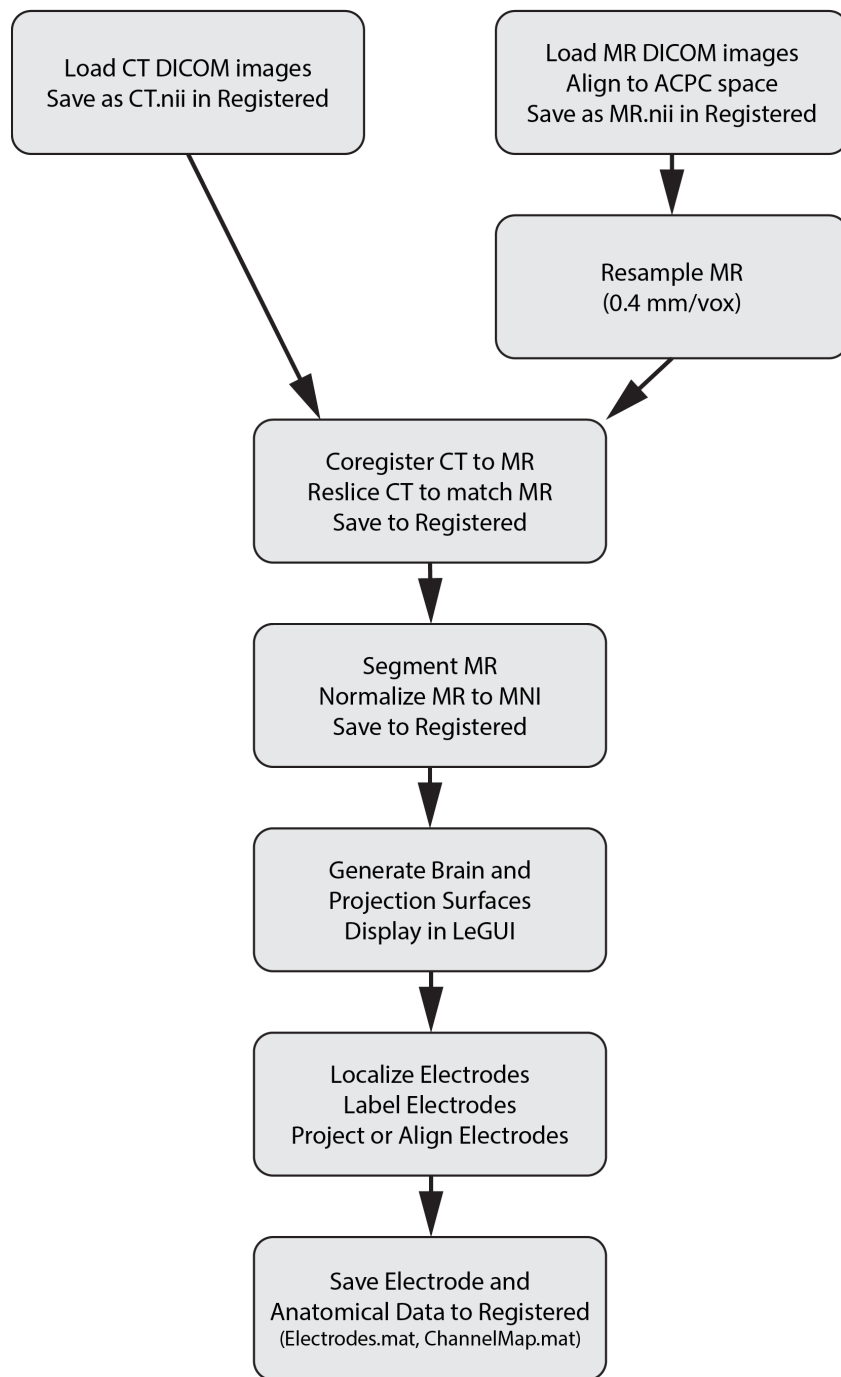

Supplementary Figure 1. Flow chart showing image processing pipeline.

Supplement: Supplementary file 1 [file Image_1.pdf]
