# Supplementary material for: LeGUI: A Fast and Accurate Graphical User Interface for Automated Detection and Anatomical Localization of Intracranial Electrodes
Source: Front Neurosci. 2021 Dec 9;15:769872. doi: 10.3389/fnins.2021.769872 (PMC8695687; doi:10.3389/fnins.2021.769872)
Supplement: Supplementary file 2 [file Table_1.docx]

Supplementary Table 1. Patient demographics and implanted electrodes

| Patient | Age | Gender | Electrode Type | Electrode Brand | Electrode Model | Electrode Hemisphere | # Of Contacts |
| --- | --- | --- | --- | --- | --- | --- | --- |
| P1 | 31 | F | ECoG | Ad-Tech | IS**R-SP10X-000, FG64C-SP10X-0CB | Right | 69 |
| P2 | 41 | F | ECoG | Ad-Tech | IS**R-SP10X-000, FG64C-SP10X-0CB | Right | 80 |
| P3 | 29 | F | ECoG + SEEG | Ad-Tech | FG64C-SP10X-0CB, DG32A-SP10X-V00, RD08R-SP05X-000 | Left | 101 |
| P4 | 45 | M | ECoG | Ad-Tech | IS04R-SP10X-000, FG64C-SP10X-0CB | Right | 55 |
| P5 | 23 | F | ECoG | Ad-Tech | IS08R-SP10X-000, IS06R-SP10X-000 | Bilateral | 104 |
| P6 | 24 | F | ECoG | Ad-Tech | IS08R-SP10X-000, FG64C-SP10X-0CB | Right | 117 |
| P7 | 41 | M | ECoG + SEEG | Ad-Tech | IS04R-SP10X-000, FG64C-SP10X-0CB, SD04R-SP05X-000 | Right | 99 |
| P8 | 38 | M | SEEG | Ad-Tech | RD**R-SP05X-000, RD10R-SP04X-000, RD10R-SP03X-000 | Bilateral | 110 |
| P9 | 22 | F | ECoG | Ad-Tech | IS**R-SP10X-000, FG64C-SP07X-0C6 | Right | 86 |
| P10 | 38 | F | SEEG | Ad-Tech | RD10R-SP05X-000 | Bilateral | 64 |
| P11 | 38 | F | SEEG | Ad-Tech | RD10R-SP05X-000 | Bilateral | 114 |
| P12 | 31 | F | SEEG | Ad-Tech | RD10R-SP05X-000 | Bilateral | 65 |
| P13 | 53 | F | SEEG | Ad-Tech | RD10R-SP05X-000 | Left | 60 |
| P14 | 40 | M | SEEG | Ad-Tech | RD10R-SP05X-000 | Bilateral | 106 |
| P15 | 34 | M | ECoG + SEEG | Ad-Tech | IS**R-SP10X-000, FG32C-SP10X-000, SD04R-SP05X-000 | Left | 89 |
| P16 | 37 | F | SEEG | Ad-Tech | RD10R-SP05X-000 | Bilateral | 95 |
| P17 | 25 | F | SEEG | Ad-Tech | RD10R-SP05X-000 | Bilateral | 93 |
| P18 | 29 | M | SEEG | Ad-Tech | RD10R-SP05X-000 | Left | 65 |
| P19 | 23 | M | SEEG | Ad-Tech | RD10R-SP05X-000 | Bilateral | 78 |
| P20 | 19 | M | SEEG | Ad-Tech | RD10R-SP05X-000 | Bilateral | 77 |
| P21 | 45 | F | SEEG | Ad-Tech | RD10R-SP05X-000 | Bilateral | 87 |
| P22 | 50 | M | SEEG | Ad-Tech | RD10R-SP05X-000 | Right | 57 |
| P23 | 24 | M | SEEG | Ad-Tech | RD10R-SP05X-000 | Bilateral | 99 |
| P24 | 26 | M | SEEG | Ad-Tech | RD10R-SP05X-000 | Bilateral | 78 |
| P25 | 26 | M | SEEG | Ad-Tech | RD10R-SP05X-000, BF08R-SP05X-000 | Bilateral | 94 |
| P26 | 56 | M | SEEG | Ad-Tech | RD10R-SP05X-000, BF08R-SP05X-000 | Bilateral | 71 |
| P27 | 47 | F | ECoG + SEEG | Ad-Tech | IS**R-SP10X-000, FG64C-MP03X-000, FG64C-SP10X-000, SD04R-SP05X-000 | Left | 136 |
| P28 | 55 | M | SEEG | Ad-Tech | RD10R-SP05X-000 | Right | 44 |
| P29 | 49 | F | SEEG | Ad-Tech | RD10R-SP05X-000 | Bilateral | 88 |
| P30 | 40 | M | SEEG | Ad-Tech | RD10R-SP05X-000, BF08R-SP05X-000 | Bilateral | 77 |
| P31 | 42 | F | SEEG | Ad-Tech | RD10R-SP05X-000, BF08R-SP05X-000, SD04R-SP05X-000 | Left | 71 |
| P32 | 53 | M | SEEG | Ad-Tech | RD10R-SP05X-000 | Bilateral | 108 |
| P33 | 38 | F | SEEG | Ad-Tech | RD10R-SP05X-000, BF08R-SP05X-000 | Bilateral | 107 |
| P34 | 34 | M | SEEG | Ad-Tech | RD10R-SP05X-000 | Right | 65 |
| P35 | 47 | M | SEEG | Ad-Tech + Dixi | Microdeep D08-****, BF08R-SP05X-000 | Bilateral | 116 |
| P36 | 25 | F | SEEG | Dixi | Microdeep D08-**** | Bilateral | 145 |
| P37 | 36 | M | SEEG | Ad-Tech | RD10R-SP05X-000, SD04R-SP05X-000 | Bilateral | 84 |
| P38 | 24 | M | SEEG | Ad-Tech | RD10R-SP05X-000, BF08R-SP05X-000, SD04R-SP05X-000 | Bilateral | 103 |
| P39 | 35 | M | SEEG | Ad-Tech + Dixi | Microdeep D08-****, BF08R-SP05X-000 | Bilateral | 130 |
| P40 | 35 | M | SEEG | Ad-Tech + Dixi | Microdeep D08-****, BF08R-SP05X-000 | Bilateral | 151 |
| P41 | 30 | F | SEEG | Dixi | Microdeep D08-**** | Bilateral | 99 |
| P42 | 50 | M | SEEG | Dixi | Microdeep D08-**** | Bilateral | 125 |
| P43 | 66 | F | SEEG | Dixi | Microdeep D08-**** | Bilateral | 155 |
| P44 | 50 | M | SEEG | Dixi | Microdeep D08-**** | Bilateral | 125 |
| P45 | 37 | F | SEEG | Ad-Tech + Dixi | Microdeep D08-****, BF08R-SP05X-000 | Bilateral | 94 |
| P46 | 21 | M | SEEG | Dixi | Microdeep D08-**** | Bilateral | 142 |
| P47 | 40 | F | SEEG | Ad-Tech + Dixi | Microdeep D08-****, BF08R-SP05X-000 | Bilateral | 168 |
| P48 | 34 | F | SEEG | Ad-Tech + Dixi | Microdeep D08-****, BF08R-SP05X-000 | Bilateral | 149 |
| P49 | 18 | M | SEEG | PMT | Depthalon 2102-**-*** | Bilateral | 126 |
| P50 | 44 | M | SEEG | PMT | Depthalon 2102-**-*** | Bilateral | 124 |
| P51 | 18 | M | SEEG | PMT | Depthalon 2102-**-*** | Left | 144 |

Asterisks indicate different model parameters for number of contacts and/or layout. These parameters do not determine contact size or primary spacing.
