# Supplementary material for: LeGUI: A Fast and Accurate Graphical User Interface for Automated Detection and Anatomical Localization of Intracranial Electrodes
Source: Front Neurosci. 2021 Dec 9;15:769872. doi: 10.3389/fnins.2021.769872 (PMC8695687; doi:10.3389/fnins.2021.769872)
Supplement: Supplementary file 3 [file Table_2.docx]

Supplementary Table 2. Electrode specifications

| Electrode Brand | Electrode Model | # Of Contacts | Contact Spacing (mm, primary) | Contact Length (mm) | Contact Diameter  (mm, exposed) | Electrode Material | Electrode Type |
| --- | --- | --- | --- | --- | --- | --- | --- |
| Ad-Tech | SD04R-SP05X-000 | 4 | 5 | 2.41 | 1.12 | Platinum/Iridium | SEEG |
| Ad-Tech | RD**R-SP05X-000 | 8-10 | 5 | 2.29 | 0.86 | Platinum/Iridium | SEEG |
| Ad-Tech | RD10R-SP04X-000, | 10 | 4 | 2.29 | 0.86 | Platinum/Iridium | SEEG |
| Ad-Tech | RD10R-SP03X-000 | 10 | 3 | 2.29 | 0.86 | Platinum/Iridium | SEEG |
| Ad-Tech | BF08R-SP05X-000 | 8 | 5 | 1.57 | 1.28 | Platinum/Iridium | SEEG |
|  |  |  |  |  |  |  |  |
| Ad-Tech | IS**R-SP10X-000 | 4-8 | 10 | N/A | 2.3 | Platinum/Iridium | ECoG |
| Ad-Tech | FG64C-MP03X-000 | 64 | 3 | N/A | 2 | Platinum/Iridium | ECoG |
| Ad-Tech | FG64C-SP10X-000 | 64 | 10 | N/A | 2.3 | Platinum/Iridium | ECoG |
| Ad-Tech | FG64C-SP07X-0C6 | 64 | 7 | N/A | 2.3 | Platinum/Iridium | ECoG |
| Ad-Tech | FG64C-SP10X-0CB | 64 | 10 | N/A | 2.3 | Platinum/Iridium | ECoG |
| Ad-Tech | DG32A-SP10X-V00 | 32 | 10 | N/A | 2.3 | Platinum/Iridium | ECoG |
|  |  |  |  |  |  |  |  |
| Dixi | Microdeep D08-10AM | 10 | 3.5 | 2 | 0.8 | Platinum/Iridium | SEEG |
| Dixi | Microdeep D08-15BM | 15 | 3.5 | 2 | 0.8 | Platinum/Iridium | SEEG |
|  |  |  |  |  |  |  |  |
| PMT | Depthalon 2102-**-*** | 8-16 | 3.5 | 2 | 0.8 | Platinum/Iridium | SEEG |

Asterisks indicate different model parameters for number of contacts and/or layout. These parameters do not determine contact size or primary spacing.
