# Supplementary material for: LeGUI: A Fast and Accurate Graphical User Interface for Automated Detection and Anatomical Localization of Intracranial Electrodes
Source: Front Neurosci. 2021 Dec 9;15:769872. doi: 10.3389/fnins.2021.769872 (PMC8695687; doi:10.3389/fnins.2021.769872)
Supplement: Supplementary file 4 [file Data_Sheet_1.docx]

**Example of the “ReadMe.txt” file generated by LeGUI**

LeGUI

Version 1.0

VersionDate 20201214

LoadTime 1490.6 sec

AssignTime 765.5 sec

Description of files generated by LeGUI

ChannelMap.mat (These variables should be used for analysis since they are mapped to recorded data channels)

* LabelMap: Cell of labels specifying electrode location and contact number (i.e. ROFC1 for right orbital frontal cortex contact 1) in 2D layout to approximate actual electrode layout

* ChannelMap1: Matrix of recorded channel numbers in 2D layout to approximate actual electrode layout

* ChannelMap2: If data was recorded using two systems this can be used for mapping 2nd system (if different)

* DepthElec: List of channel numbers from either ChannelMap1 or 2 that represent depth electrodes

* MicroElec: List of channel numbers from either ChannelMap1 or 2 that represent micro electrodes

* GndElec: List of channel numbers from either ChannelMap1 or 2 that represent ground electrodes

* RefElec: List of channel numbers from either ChannelMap1 or 2 that represent reference electrodes

* AtlasNames: Cell of atlas names that correspond to the columns of ElecAtlasProj

* ElecAtlasProj: Cell of atlas labels where rows represent recorded channel and columns represent a specific atlas

* ElecAtlasProbProj: Cell of atlas label probabilities taken from a 1cm search radius where rows represent recorded channel and columns represent results for a specific atlas

* ElecTypeProj: Cell of gray/white/unknown labels where rows represent recorded channel

* ElecCOMIdxProj: Matrix of voxel indices (row/col/page) that represent the center-of-mass of each electrode where rows represent recorded channel

* ElecFullIdxProj: Cell of voxel indices that represent the volume of each electrode where rows represent recorded channel

* ElecXYZProj: Matrix of electrode locations (x/y/z) in patient world space (units mm) where rows represent recorded channel

* ElecXYZMNIProj: Matrix of electrode locations in standard MNI space (units mm) where rows represent recorded channel

Electrodes.mat (These variables are used by the GUI and are organized by draw-order of electrodes)

* ElecMapRaw: Cell where rows represent electrode draw-order and columns represent values from LabelMap, ChannelMap1, and ChannelMap2

* DepthElecRaw: List of depth electrodes numbered by corresponding row in ElecMapRaw

* MicroElecRaw: List of micro electrodes numbered by corresponding row in ElecMapRaw

* GndElecRaw: List of ground electrodes numbered by corresponding row in ElecMapRaw

* RefElecRaw: List of reference electrodes numbered by corresponding row in ElecMapRaw

* AtlasNames: Cell of atlas names that correspond to the columns of ElecAtlasRaw and ElecAtlasProjRaw

* ElecAtlasRaw: Cell of atlas labels where rows represent electrode draw-order and columns represent a specific atlas

* ElecAtlasProjRaw: Projected version of ElecAtlasRaw

* ElecAtlasProbProjRaw: Cell of atlas label probabilities taken from a 1cm search radius where rows represent electrode draw-order and columns represent results for a specific atlas

* ElecTypeRaw: Cell of gray/white/unknown labels where rows represent electrode draw-order

* ElecTypeProjRaw: Projected version of ElecTypeRaw

* ElecCOMIdxRaw: Matrix of voxel indices (row/col/page) that represent the center-of-mass of each electrode where rows represent electrode draw-order

* ElecCOMIdxProjRaw: Projected version of ElecCOMIdxRaw

* ElecFullIdxRaw: Cell of voxel indices that represent the volume of each electrode where rows represent electrode draw-order

* ElecFullIdxProjRaw: Projected version of ElecFullIdxRaw

* ElecXYZRaw: Matrix of electrode locations (x/y/z) in patient world space (units mm) where rows represent electrode draw-order

* ElecXYZProjRaw: Projected version of ElecXYZRaw

* ElecXYZMNIRaw: Matrix of electrode locations in standard MNI space (units mm) where rows represent electrode draw-order

* ElecXYZMNIProjRaw: Projected version of ElecXYZMNIRaw

Surfaces.mat

* BrainSurfRaw: Faces/vertices for brain surface

* ProjSurfRaw: Faces/vertices for a highly smoothed brain surface that is used to define vectors and project electrodes

ACPCmm.mat: Matrix where rows represent x/y/z coordinates of AC, PC, and superior mid-saggital points in patient world space

MR_coreg.mat: Coregistration results of CT to MR image generated by SPM12

MR_seg8.mat: Segmentation results of MR generated by SPM12

CT.nii: CT image coregistered and resliced to match MR.nii

MR.nii: MR image aligned to mid-ACPC coordinate system

MRWhite.nii: White matter segmentation results from SPM12

MRGray.nii: Gray matter segmentation results from SPM12

MRCSF.nii: CSF segmentation results from SPM12

MRBone.nii: Skull segmentation results from SPM12

MRSkin.nii: Skin segmentation results from SPM12

iy_MR.nii: Inverse deformation matrices from patient to MNI space

y_MR.nii: Deformation matrices from MNI to patient space

lw*.nii: All files with prefix "lw" are atlases deformed to patient space for viewing in GUI and for analysis (labels are preserved).
