# Supplementary material for: LeGUI: A Fast and Accurate Graphical User Interface for Automated Detection and Anatomical Localization of Intracranial Electrodes
Source: Front Neurosci. 2021 Dec 9;15:769872. doi: 10.3389/fnins.2021.769872 (PMC8695687; doi:10.3389/fnins.2021.769872)
Supplement: Supplementary file 5 [file Data_Sheet_2.docx]

**Supplementary Methods**

*Installation requirements*

LeGUI can be run from the source code using a recent version of MATLAB (R2019b or newer) with the Image Processing, Parallel Computing, and Statistics and Machine Learning toolboxes installed. All other functions including the Statistical Parametric Mapping (SPM12) functions for the core image processing steps are included in the source. LeGUI can also be run from executables that are compiled for Windows, Mac, and Linux operating systems. The only requirement to run the executables is to download and install the appropriate version of MATLAB Runtime (<https://www.mathworks.com/products/compiler/matlab-runtime.html>). All other dependencies are packaged with the executables. The source code and executables can be downloaded at <https://github.com/Rolston-Lab/LeGUI/releases/latest#user-content-downloads>. For more details, links to an installation guide and user manual are available on the GitHub main page (<https://github.com/Rolston-Lab/LeGUI>).

*2D visualizations*

The 2D display on the left of LeGUI shows the overlaid slice planes for the coregistered MRI and CT images (Figure 1B). The mouse scroll wheel or left and right arrows on the keyboard can be used to scroll through each slice. Initially, the CT is not visible; however, the MR can be faded out of view to reveal the CT using the slider bar at the bottom. Fading the MR with respect to the CT is a convenient way to visually check the accuracy of the coregistration step, as well as to visualize and manually localize electrodes. In addition, the orientation of the slice plane can be changed from the default “sagittal” view to either a “coronal” or “axial” view. The radiological convention for image orientation is used for the axial view where the patient’s left is displayed on the right in the image and vice versa. Image brightness and contrast can be adjusted with a left drag over the image or by changing the values in the “CLow” and “CHigh” edit boxes. After electrodes have been localized, they will be displayed as embedded blue dots. These dots appear in both the MRI and CT views and are drawn to scale based on the specified electrode radius. A left click near an electrode will select it, and its color will change from blue to red. Pressing “Shift” on the keyboard and left clicking the mouse can be used to select multiple electrodes at once. Selecting an electrode in the 2D plot will also select and highlight the corresponding electrode in the 3D plot. This “linking” of the 2D and 3D plots during electrode selection provides an efficient way to explore the electrode space and visualize nearby MR structures. The location of a selected electrode is displayed in the upper left “Electrode Location” panel of LeGUI. This shows location relative to the chosen atlas, as well as electrode label, channel number, and gray or white classification. Label and channel number are only available after assigning these values using the electrode assignment interface. Additionally, selected electrodes can be deleted by pressing “Delete” on the keyboard. A toolbar in the upper left corner of the plot can be used to activate tools for zooming or panning the image or saving a snapshot of the image as a portable network graphics file to disk.

*3D visualizations*

The 3D display on the right of LeGUI shows brain and projection surfaces, as well as electrode locations (Figure 1B). Initially, the brain surface is visible, and the projection surface is hidden from view. Visibility for each surface can be changed from a value of 0 (invisible) to values of 0.1, 0.2, 0.5, or 1 (visible) using controls at the bottom of the display. The projection surface is used as a boundary for repositioning ECoG electrodes that have moved because of brain shift from a craniotomy. It is sometimes useful to visualize this surface while performing the projections. In addition to the surfaces, the 3D locations of electrodes are also displayed as blue dots. To view the electrodes, the visibility of the brain surface can be decreased to values less than 1. A left click near an electrode will select it, and its color will change from blue to red. Selecting an electrode in the 3D display will also select and highlight the corresponding electrode in the 2D display. Multiple selections can be made by pressing “Shift” on the keyboard and left clicking the mouse, and deletions can be made by pressing the “Delete” key, as in the 2D display. A toolbar in the upper left corner contains tools to zoom, pan, and rotate the view. This toolbar also contains a polygon tool (“plus” symbol) that can be used to select multiple electrodes with a left drag of the mouse to circumscribe a group of electrodes. Similar to the 2D display, a snapshot of the current view can be exported to disk as a portable network graphics (PNG) file.

*Electrode labeling and channel assignment*

The “Assign Electrodes” button in LeGUI will open a user interface for assigning labels and channel numbers (Figure 1C, left). This interface consists of a 3D display of the brain surface and electrode locations on the left and a table with labels and channel numbers on the right. Initially, labels are added to the table in a 2D layout that approximates the 3D electrode locations. For SEEG, this typically consists of a column in the table for each lead with the deepest electrodes as the bottom row and left and right leads separated by an empty column filled with not-a-numbers (NaNs). Labels in each column typically consist of one or more letters describing the general location within the brain followed by a number that represents the electrode (or contact) position. For example, a right hippocampal SEEG lead with 10 electrodes (contacts) would be represented by a column of labels starting with “RHIP1” as the bottom row and ending with “RHIP10” as the top row. ECoG grids and strips can be labeled in a similar fashion. Columns (or rows) of “NaN” can be used to separate the individual components such as different leads for SEEG or different grids/strips for ECoG. This separation method is optional and is primarily used to provide visual clarity to the layout. Labels are entered into the table by selecting a cell and typing the label. Once a label has been entered, other labels in a column (or row) can be autopopulated by pressing Ctrl + an arrow key. The direction of the arrow indicates the direction the cells will be auto populated relative to the currently selected cell. For example, if “RHIP1” has been entered in the lower-left cell of the table and it is selected (i.e., red), pressing Ctrl+up-arrow on the keyboard will fill the remaining cells in that column with “RHIP” followed by increasing values from 2 to 10 (i.e., RHIP2, RHIP3, …, RHIP10). Likewise, pressing Ctrl+Alt+up-arrow will fill with decreasing values.

Similar to labels, channel numbers can be added to the table that indicate the link between electrode and corresponding channel of recorded data from the clinical (or research) recording equipment. The process of adding channels to the table is the same as adding labels. However, channels must consist of integers and cannot contain any alphanumeric characters like the labels. To add channels, select either “ChannelMap 1” or “ChannelMap 2” from the drop-down menu, select a cell in the table, and type the desired integer value. Auto population of columns or rows with increasing (or decreasing) channel values is available as described above.

After labels have been added to the table for all electrodes, assignments between electrode and label can be made. This process involves selecting electrodes in the 3D display, selecting the corresponding labels in the table, and pressing the “Assign” button. If multiple electrodes have been selected, a basic algorithm has been employed to estimate the order of assignment within that group of electrodes. Multiple selected 3D electrodes are sorted in descending order four different ways: distance from mid-commissural point (0,0), value along the x-axis, value along the y-axis, and value along the z-axis. The average distance between neighboring electrodes is then computed for each sort type, and the sort type with the minimum average distance is chosen as the electrode order. Multiple selected labels in the table are ordered from top-to-bottom and left-to-right by default in Matlab. The sorted electrodes and corresponding selected labels in the table are then assigned one-to-one based on their respective ordering. This semiautomatic assignment procedure works best for SEEG leads that are represented as separate columns in the table; however, the same principles can be applied when assigning labels to ECoG grids and strips.

Color codes are used when making electrode assignments to help clarify the assignment type and make it easy to identify electrodes that have not received an assignment. Electrodes are colored blue in the 3D display, and labels are uncolored in the table if no assignment has been made. After assignment, SEEG (depth) electrodes appear black and ECoG (surface) electrodes appear green in the 3D display. Likewise, cells in the table that have been assigned to depth electrodes are black, and those assigned to surface electrodes are green. Additionally, the color pink is used when assigning microelectrodes. Selected electrodes or cells in a table are always colored red.

After all assignments have been made, there is an option to check those assignments to verify accuracy. This mode can be enabled by selecting “Check” from the drop-down menu. Once enabled, selecting an assigned electrode in the 3D display will also select (highlight) the corresponding label in the table. Likewise, selecting an assigned label in the table will also select (highlight) the corresponding electrode in the 3D display. An efficient method of verifying all assignments involves selecting a label in the table and using the arrows on the keyboard to move across labels while simultaneously watching the highlighted electrodes move in the 3D display.

After checking assignments, the assignment window can be closed. Closing the window will send all assignments back to LeGUI and populate the main 3D display with the new colormap.

*Custom colormaps*

The ability to load custom colormaps to color electrodes in the 3D display in LeGUI has been included. This can be used to visualize features of recorded electrophysiology data relative to electrode location such as LFP power, seizure-onset estimations, or evoked potentials during stimulation. An example of this can be seen in Figure 6 where the amplitude of corticocortical evoked potentials is shown as a colormap applied to the 3D electrodes. To use this feature, electrodes must be assigned a channel number that corresponds to the recorded data channel in the clinical or research recordings. A Matlab file must then be loaded that contains a list of numbers ranging from zero to one that is equal in length to the max number of recorded data channels. Once loaded, the “custom” colormap can be selected from the dropdown menu.
